# Inhibiting NR5A2 targets stemness in pancreatic cancer by disrupting SOX2/MYC signaling and restoring chemosensitivity

**DOI:** 10.1186/s13046-023-02883-y

**Published:** 2023-11-28

**Authors:** Quan Zheng, Jiajia Tang, Alexandra Aicher, Tony Bou Kheir, Berina Sabanovic, Preeta Ananthanarayanan, Chiara Reina, Minchun Chen, Jian-Min Gu, Bin He, Sonia Alcala, Diana Behrens, Rita T. Lawlo, Aldo Scarpa, Manuel Hidalgo, Bruno Sainz, Patricia Sancho, Christopher Heeschen

**Affiliations:** 1 Center for Single-Cell Omics, School of Public Health, Shanghai Jiao Tong University School of Medicine, Shanghai, 200025 China; 2 State Key Laboratory of Systems Medicine for Cancer, Shanghai Jiao Tong University School of Medicine, Shanghai, China; 3 Precision Immunotherapy, Graduate Institute of Biomedical Sciences, China Medical University, Taichung, Taiwan; 4 Immunology Research and Development Center, China Medical University, Taichung, Taiwan; 5 Barts Cancer Institute, Queen Mary University of London, London, UK; 6 Pancreatic Cancer Heterogeneity Lab, Candiolo Cancer Institute - FPO - IRCCS, Candiolo, Turin, Italy; 7 Department of Thoracic Surgery, Zhongshan Hospital, Fudan University, Shanghai, China; 8 National Engineering Research Center for Biomaterials, Sichuan University, Chengdu, China; 9 Molecular Pathology Programme, Spanish National Cancer Research Centre (CNIO), Madrid, Spain; 10 Instituto de Investigaciones Biomédicas “Alberto Sols” CSIC-UAM, Chronic Diseases and Cancer Area 3 Instituto Ramón y Cajal de Investigación Sanitaria (IRYCIS), Centro de Investigación Biomédica en Red, Área Cáncer, CIBERONC, ISCIII, Madrid, Spain; 11 Experimental Pharmacology and Oncology Berlin-Buch GmbH, Berlin, Germany; 12 Department of Diagnostics and Public Health, Section of Pathology, University of Verona, Verona, Italy; 13 ARC-Net, Applied Research On Cancer Centre, University of Verona, Verona, Italy; 14 Clinical Research Programme, Spanish National Cancer Research Centre (CNIO), Madrid, Spain; 15 IIS Aragon, Hospital Universitario Miguel Servet, 50009 Saragossa, Spain

**Keywords:** Pancreatic ductal adenocarcinoma, Cancer stem cells, Metabolism, SOX2, MYC

## Abstract

**Background:**

Pancreatic ductal adenocarcinoma (PDAC) is a profoundly aggressive and fatal cancer. One of the key factors defining its aggressiveness and resilience against chemotherapy is the existence of cancer stem cells (CSCs). The important task of discovering upstream regulators of stemness that are amenable for targeting in PDAC is essential for the advancement of more potent therapeutic approaches. In this study, we sought to elucidate the function of the nuclear receptor subfamily 5, group A, member 2 (NR5A2) in the context of pancreatic CSCs.

**Methods:**

We modeled human PDAC using primary PDAC cells and CSC-enriched sphere cultures. NR5A2 was genetically silenced or inhibited with Cpd3. Assays included RNA-seq, sphere/colony formation, cell viability/toxicity, real-time PCR, western blot, immunofluorescence, ChIP, CUT&Tag, XF Analysis, lactate production, and in vivo tumorigenicity assays. PDAC models from 18 patients were treated with Cpd3-loaded nanocarriers.

**Results:**

Our findings demonstrate that NR5A2 plays a dual role in PDAC. In differentiated cancer cells, NR5A2 promotes cell proliferation by inhibiting CDKN1A. On the other hand, in the CSC population, NR5A2 enhances stemness by upregulating SOX2 through direct binding to its promotor/enhancer region. Additionally, NR5A2 suppresses MYC, leading to the activation of the mitochondrial biogenesis factor PPARGC1A and a shift in metabolism towards oxidative phosphorylation, which is a crucial feature of stemness in PDAC. Importantly, our study shows that the specific NR5A2 inhibitor, Cpd3, sensitizes a significant fraction of PDAC models derived from 18 patients to standard chemotherapy. This treatment approach results in durable remissions and long-term survival. Furthermore, we demonstrate that the expression levels of NR5A2/SOX2 can predict the response to treatment.

**Conclusions:**

The findings of our study highlight the cell context-dependent effects of NR5A2 in PDAC. We have identified a novel pharmacological strategy to modulate SOX2 and MYC levels, which disrupts stemness and prevents relapse in this deadly disease. These insights provide valuable information for the development of targeted therapies for PDAC, offering new hope for improved patient outcomes.

**Graphical Abstract:**

**A** Schematic illustration of the role of NR5A2 in cancer stem cells versus differentiated cancer cells, along with the action of the NR5A2 inhibitor Cpd3. **B** Overall survival of tumor-bearing mice following allocated treatment. A total of 18 PDX models were treated using a 2 x 1 x 1 approach (two animals per model per treatment); n=36 per group (illustration created with biorender.com).

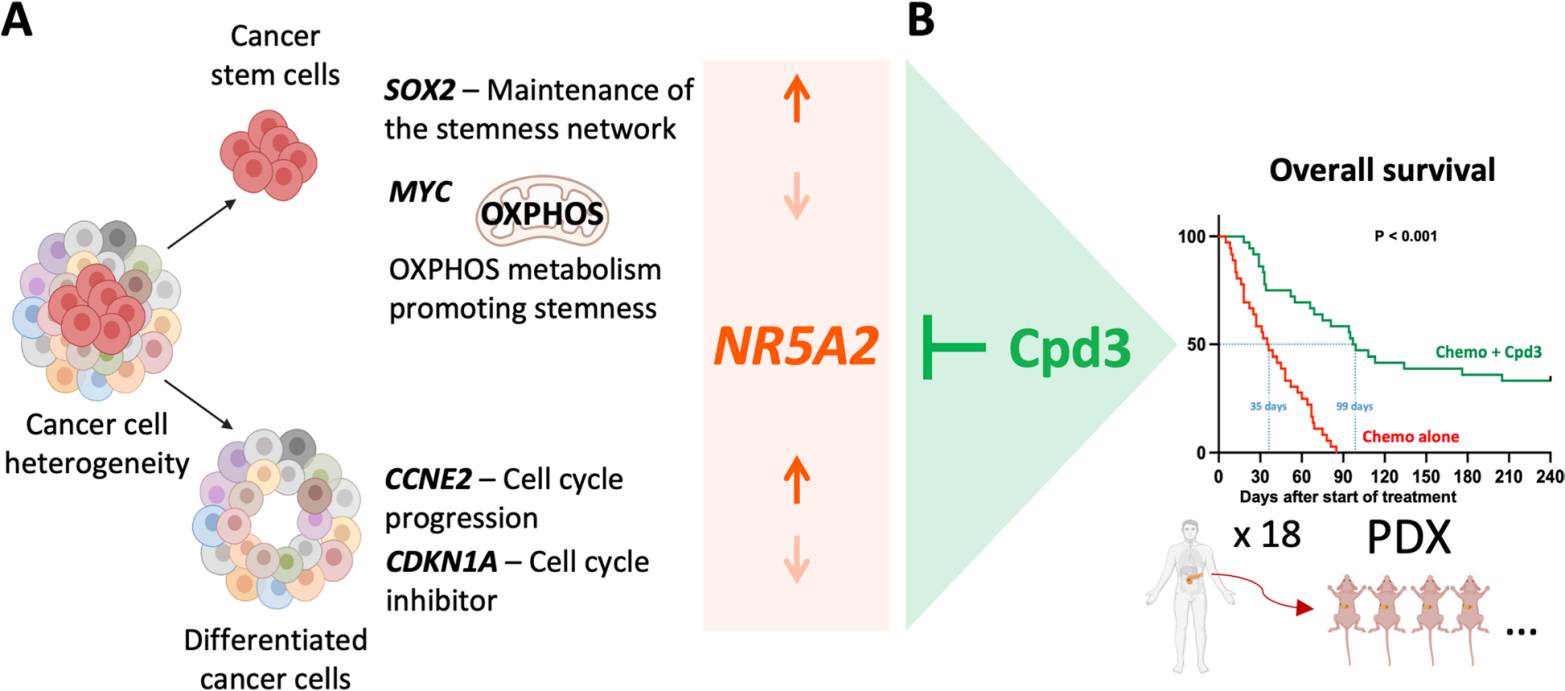

**Supplementary Information:**

The online version contains supplementary material available at 10.1186/s13046-023-02883-y.

## Statement of significance

The present study provides conclusive evidence that NR5A2 is a suitable target for pancreatic cancer via a dual mechanism involving SOX2 and MYC and that a considerable fraction of PDAC tumors responded to NR5A2 inhibition, but a combination with tumor-debulking chemotherapy is needed for improved outcomes. These findings bear the potential to eventually improve the outcomes of pancreatic cancer patients.

## Introduction

Pancreatic cancer, including pancreatic ductal adenocarcinoma (PDAC) as the most common type, is highly lethal due to extensive metastasis [1, 2], and current treatments rarely result in long-term survival [3–5]. By 2030, PDAC might become the 2^nd^ most frequent cause of cancer-related death [6]. Compelling evidence, from our lab and others, confirms the presence of stem cell-like cells in PDAC [7–9]. These cells can be recognized based on the surface expression of CD133 either independently or in conjunction with CXCR4 [7] or CD44 [10]. These cells, even at a single-cell level, are uniquely capable of propagating the tumor, much like normal stem cells fuel proliferation and differentiation in normal tissue and are therefore termed cancer stem cells (CSC). Developing effective and readily translatable CSC-targeting treatments for the clinic will require a thorough understanding of the regulatory machinery of CSCs and the identification of means to specifically disrupt the cancer stemness network without affecting normal stem cells.

For this purpose, we performed an exploratory RNA-seq analysis of CSCs versus their differentiated progenies using a representative set of PDAC patients. Our data uncovered a marked overexpression of the regulatory molecule *NR5A2* in pancreatic CSCs versus their differentiated progenies. This discovery came as a surprise because previous research had indicated that NR5A2 is promoting PDAC cell proliferation [11], and it is widely known that the proliferation rate of pancreatic CSCs is notably lower when compared to their differentiated PDAC cell counterparts [12]. NR5A2 acts as a ligand-dependent transcription factor and plays a multifaceted role in both development and disease. During early embryonic development, NR5A2 is involved in the differentiation of key organs like the liver, intestine, and pancreas [13], but in adults, it regulates steroidogenesis and cholesterol/bile acid homeostasis [14]. NR5A2 also maintains pluripotent stem cells and influences key transcription factors like Oct4 and Nanog [15]. In gastrointestinal tumors, NR5A2 has been shown to interact with the Wnt/β-catenin signaling pathway to promote cell proliferation and self-renewal, potentially contributing to colorectal cancer. In previous studies using immortalized cancer cell lines, *NR5A2* was shown to regulate the proliferation of bulk pancreatic cancer cells [16]. In the adult pancreas, NR5A2 is essential for normal pancreatic function, while in pancreatic cancer, its role is more complex, with evidence suggesting it can both promote and inhibit cancer progression [17]. Therefore, we performed a comprehensive study to define the cancer cell phenotype-specific role of NR5A2 in PDAC and explored its potential as a therapeutic target in a large-scale preclinical study. A pharmacological inhibitor of *NR5A2* (Cpd3), first described in 2013, specifically binds to the ligand binding domain of NR5A2 and inhibits the transcriptional activity of NR5A2 both in vitro [18, 19] and in vivo in a zebrafish model [20]. In this study, we investigated the effects of NR5A2 inhibition using the pharmacological inhibitor Cpd3. By targeting NR5A2, we aimed to disrupt the cancer stemness network and evaluate its potential as a therapeutic strategy for PDAC.

## Results

### *NR5A2* is overexpressed in pancreatic CSCs

**Fig. 1:**
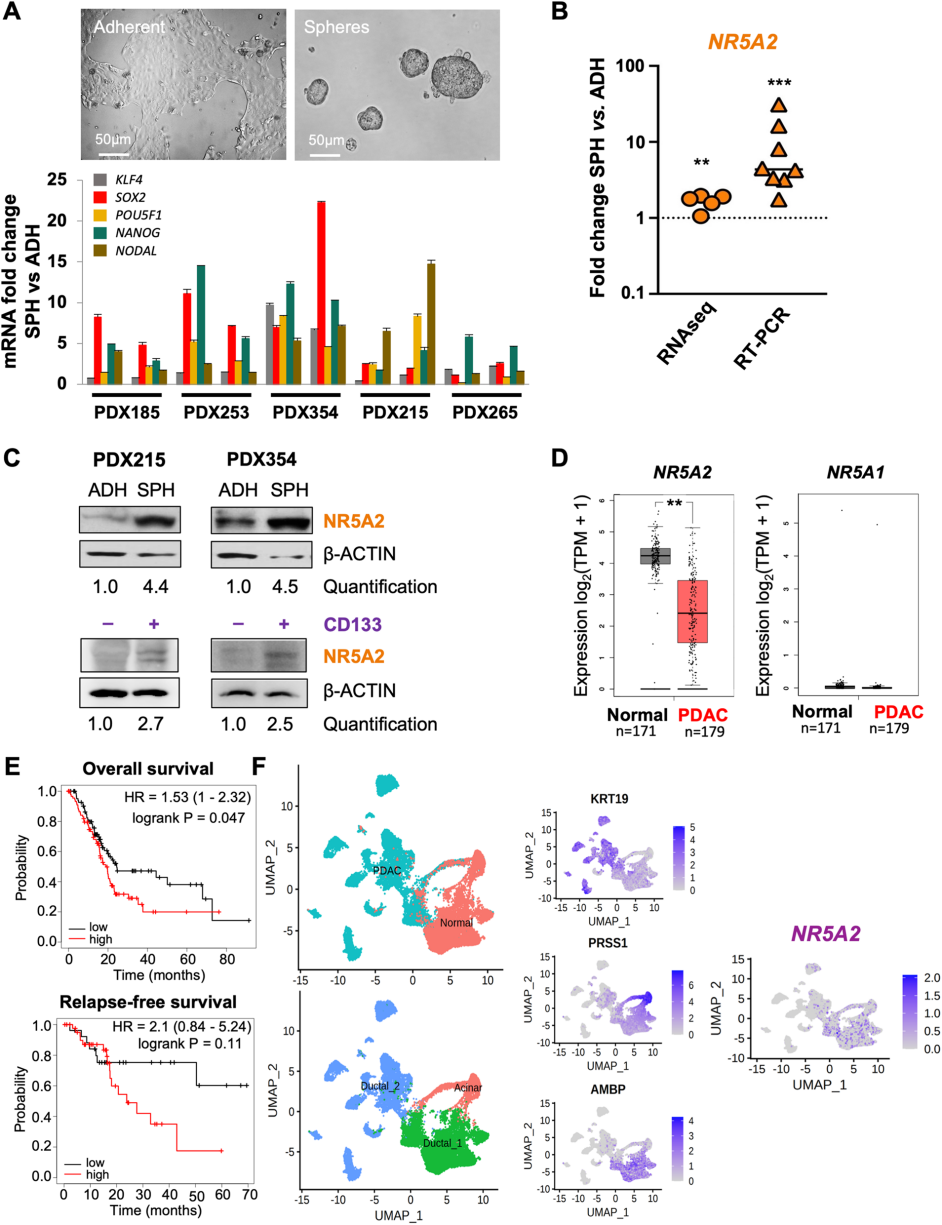
*NR5A2* is overexpressed in pancreatic cancer stem cells*.* **A** Upregulation of stemness-related mRNAs in CSC-enriched sphere (SPH) versus adherent (ADH) cultures derived from a diverse set of primary PDAC tumors. Representative images of adherent and sphere cultures are shown in the upper panel. The lower panel represents the quantification of mRNA expression. Each sample was analyzed in biological duplicates, which are displayed side by side. The error bars indicate the mean ± standard deviation (SD) from technical duplicates. **B** RNA sequencing and qPCR validation for *NR5A2* expression expressed as fold-change for adherent (ADH) versus sphere (SPH) cultures. The dotted line indicates reference levels for adherent cells, set as 1.0. RNA sequencing data are displayed as pooled data from *n* = 5 different PDAC cultures using biological duplicates. The qPCR validation was performed from pooled data derived from *n* = 8 different PDAC cultures. **C** Western blot analysis for NR5A2 protein levels in adherent (ADH) versus sphere (SPH) cultures in PDX215 and PDX354 cells (upper panel), as well as in CD133 + versus CD133– sorted PDAC cultures (lower panel). β-Actin was used as the loading control. **D** *NR5A2* and *NR5A1* mRNA expression in PDAC versus normal tissue as analyzed by GEPIA 2. **E** Prognostic significance of NR5A2 mRNA expression levels for overall survival (OS) and relapse-free survival (RFS) for PDAC as analyzed by Kaplan–Meier Plotter databases. **F** UMAP projections of single-cell RNA-seq data consisting of 24 PDAC tumor samples and 11 control pancreases without any treatment, as described by Peng et al. [24]. The UMAP plots illustrate the clustering of PDAC tumor samples into two distinct groups: cancer cells versus normal cells (left, upper panel) and ductal cells versus acinar cells (left, lower panel). Ductal cells were characterized by the expression of KRT19 and AMBP, while acinar cells exhibited high expression of PRSS1. The UMAP plot on the right visualizes the distribution of NR5A2 expression within the ductal and acinar cell clusters. Asterisks indicate significance at the indicated levels: ** *p* < 0.01, *** *p* < 0.001. Please also see Supplementary Fig. 1

To uncover candidate genes that may play a role in governing stemness in PDAC, we conducted RNA-seq (E-MTAB-3808) on CSC-enriched anchorage-independent sphere cultures in comparison to adherent cultures. We employed a representative selection of five validated PDAC models [21–23] to pinpoint genes that exhibit differential regulation within the CSC subset (Fig. 1A, upper panel). Successful enrichment for CSCs was demonstrated by increased expression of stemness-associated genes (Fig. 1A, lower panel). Interestingly, *NR5A2* was among the most significantly and consistently upregulated genes (Figs. 1B and S1A). The RNA-seq data could be validated by qPCR using a total of 8 PDX models showing a consistent and significant upregulation of *NR5A2* by up to 32-fold in spheres relative to their differentiated counterparts (Fig. 1B). In line with these mRNA data, we found detectable NR5A2 protein levels in differentiated cancer cells. However, NR5A2 expression was further increased in CSCs following anchorage-independent sphere culture or CD133 sorting (Figs. 1C & S1B). These data suggested a specific functional role for *NR5A2* in pancreatic CSCs.

Notably, using the GEPIA 2 database (http://gepia.cancer-pku.cn/index.html), *NR5A2* was found to be most strongly expressed in normal pancreas tissue but downregulated in corresponding PDAC tissue, whereas NR5A1 was not detectable in either tissue (Fig. 1D). *NR5A2* mRNA expression was also more strongly expressed in normal liver and bile duct, but no change in expression was observed for the respective cancer tissues (Fig. S1C). All other tissues showed the expected low *NR5A2* mRNA expression. When stratifying patients for *NR5A2* expression in their respective PDAC tissue, patients with increased *NR5A2* expression showed reduced overall survival and a trend towards diminished relapse-free survival (Fig. 1E). Consistent results were obtained using the ArrayExpress database E-MTAB-1791 (Fig. S1D) [25]. Expectedly, no such differences could be observed for other cancer types, such as lung, breast, and bladder cancer (Fig. S1E) due to low *NR5A2* expression levels (Fig. S1B). Single-cell RNA-seq of PDAC tissue confirmed that *NR5A2* is expressed more prominently in non-transformed acinar and ductal cells in the pancreas compared to transformed PDAC cells (Figs. 1F & S1F) [24, 26]. Notably, however, we could still identify some transformed ductal cells that showed detectable *NR5A2* expression, suggesting considerable intratumoral heterogeneity for *NR5A2* expression.

### *NR5A2* regulates proliferation of differentiated PDAC cells

As *NR5A2* expression was rather modest in our differentiated PDAC cells compared to CSCs, we first aimed to validate above findings in our primary PDAC models. For this purpose, we explored a pharmacological inhibitor of *NR5A2* (Cpd3) to inhibit the activity of the NR5A2 protein and assessed the treatment effects of Cpd3 in PDAC by tracking *NR5A2* mRNA levels. Using three different primary PDAC cultures, Cpd3 showed no acute or unspecific toxicity at 24 h as evidenced by cell viability and toxicity assays (Fig. S2A-B). Still, we observed a substantial reduction in *NR5A2* mRNA levels following a single-shot treatment as early as 24 h after drug administration, and the reduction was maintained for at least 72 h (Fig. S2C-D). Consistently, NR5A2 protein levels were also reduced by ~ 50% (Fig. S2E).

**Fig. 2:**
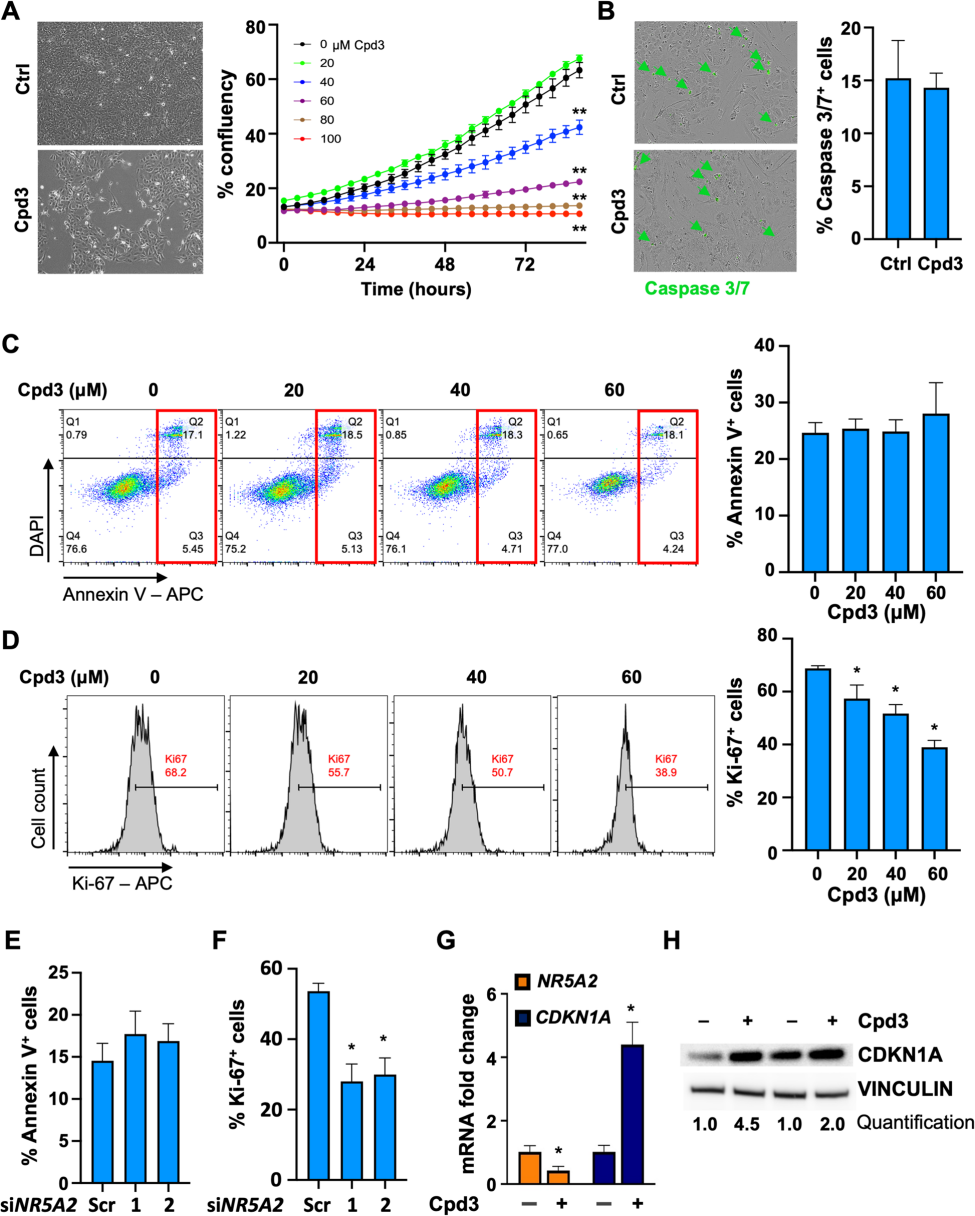
*NR5A2* controls proliferation in differentiated cancer cells and is drugable*.* **A** Cell density and morphology were assessed after 72 h of treatment with Cpd3 (40 µM) versus control (Ctrl). Representative results for PDX215 cultures are shown (left). Additionally, the overall cell confluency was monitored over an 80-h period using the IncuCyte^®^ platform (right). **B** Caspase 3/7 staining for cells treated with control or Cpd3 (40 µM) for 72 h. Quantification and representative images for PDX215 from *n* = 5 experiments are presented. **C** Apoptosis analysis was performed using DAPI/Annexin V flow cytometry in PDX215 cells treated with graded doses of Cpd3. The lower right quadrant indicates early apoptosis, while the upper right quadrant represents late apoptosis (marked by the red rectangle). Quantification reveals the percentage of combined early and late apoptotic cells (*n* = 3). **D** Flow cytometric dot blot analyses were carried out to examine Ki-67 expression after 72 h of treatment with graded doses of Cpd3 in PDX215 cells. The quantification depicts the percentage of Ki-67^+^ cells (*n* = 3). **E** Apoptosis analysis, measured by DAPI/Annexin V flow cytometry, and the measurement of proliferation (**F**), indicated by the number of Ki67^+^ cells, were conducted in PDX215 cells treated with scramble siRNA and the two most effective si*NR5A2* variants (#1 and #2) for 72 h. Quantification of four biological replicates is displayed. **G** qPCR fold change of *NR5A2* and *CKDN1A* (p21) mRNA following 72-h treatment with Cpd3 (80 µM) in PDX215 cells. **H** Western blot analysis of CDKN1A protein levels following 72 h treatment with Cpd3 (80 µM) in PDX215 cells. Data were presented as mean ± SD and statistically analyzed using two-tailed Mann–Whitney tests. Asterisks indicate significance at the indicated levels: * *p* < 0.05 and ** *p* < 0.01. Please also see Supplementary Fig. 2

Next, we treated differentiated PDAC cells with graded doses of Cpd3 and monitored cell confluency on the Incucyte® platform. Our results showed a decrease in cell confluency with increasing doses of Cpd3 (Figs. 2A & S2F). Notably, Caspase 3/7 immunofluorescence (Fig. 2B & S2G) and AnnexinV flow cytometry (Figs. 2C & S2H-I) revealed no change in the apoptotic rate of the treated PDAC cells after 72 h across the relevant pharmacological range of Cpd3 concentrations. However, we noted a dose-dependent reduction in cell proliferation, as evidenced by flow cytometry using the proliferation marker Ki-67 (Figs. 2D & S2J-K). These findings for pharmacological inhibition of NR5A2 could be further validated using two effective siRNA against *NR5A2* (Figs. 2E-F & S2L-M), which corroborated the lack of change in apoptotic cells and the reduction in Ki-67^+^ proliferative cells after si*NR5A2* silencing. In line with these findings, Cpd3 treatment resulted in a marked downregulation of mRNA expression for *CCNE2* (G1 cyclin binding *CDK2*) and upregulation of *CDKN1A (*cyclin-dependent kinase inhibitor 1; p21*)*, respectively (Figs. 2G & S2N). These changes were accompanied by an increase in CDKN1A (p21) protein levels (Fig. 2H). These results indicate that NR5A2 inhibition specifically affects cell proliferation in differentiated PDAC cells without inducing apoptosis or cell death. Based on these observations, we determined that a Cpd3 dose range of 20-80 μM was suitable for subsequent experiments.

### *NR5A2* controls stemness in PDAC

**Fig. 3:**
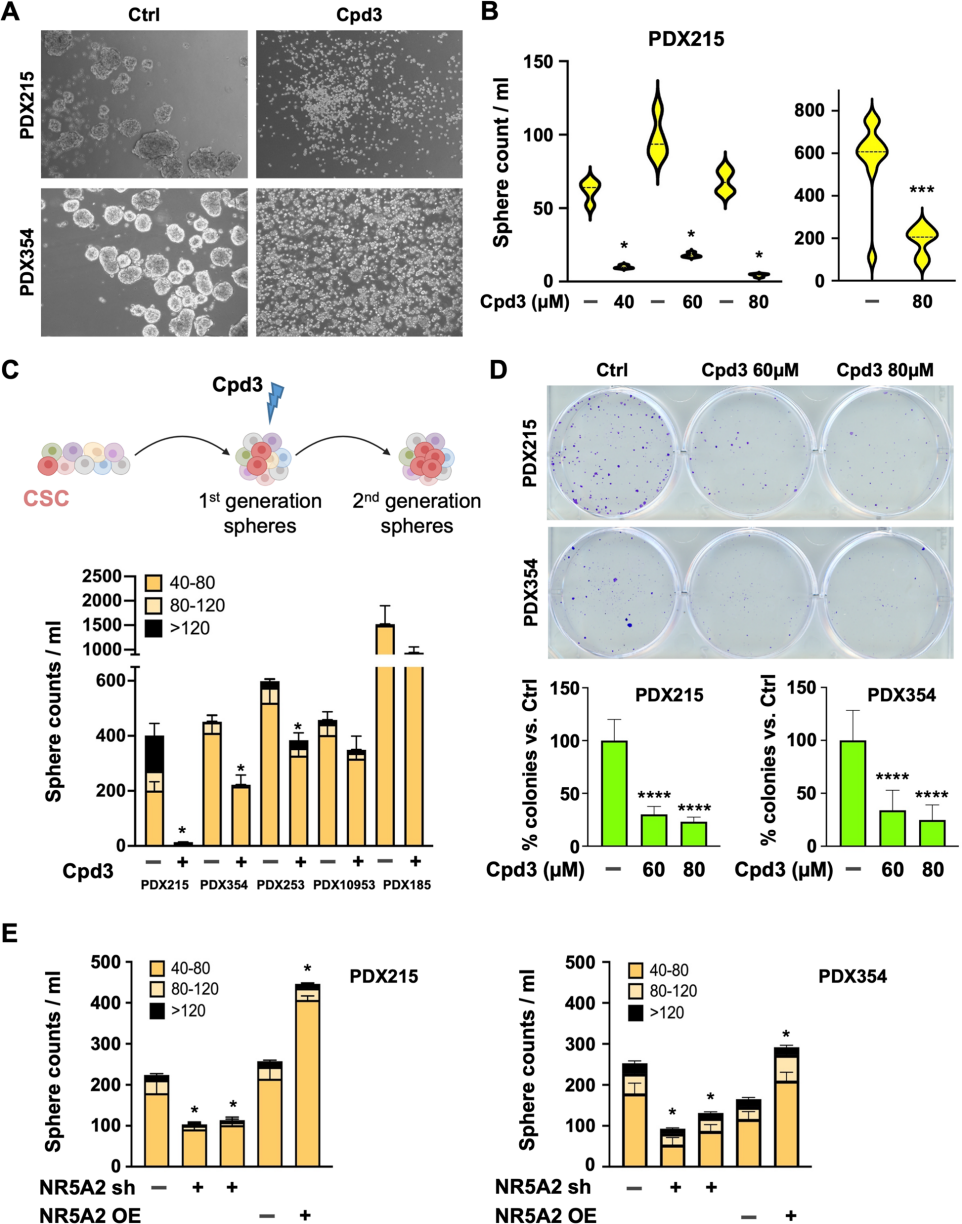
*NR5A2* controls stemness in PDAC*.* **A** Sphere formation capacity on day 7 following Cpd3 treatment. Representative images for PDX215 and PDX354 following treatment with Cpd3 (60 µM). **B** Quantification of primary sphere formation capacity on day 7 of Cpd3 treatment for PDX215 (*n* = 4 biological replicates) and PDX354 (*n* = 10 biological replicates). Data are shown as violin plots with dotted lines indicating the median. **C** The schematic (created with biorender.com) illustrates the process of forming first-generation spheres over 7 days. This is succeeded by the disintegration of the formed spheres into a single-cell suspension, and then the commencement of second-generation spheres over another 7-day period, with or without Cpd3 treatment (upper panel). The lower panel shows the secondary sphere formation of cells treated with Cpd3 (60 µM) for five different PDAC cultures. **D** Colony formation capacity of cells treated with Cpd3 for 72 h. Representative pictures (top), quantification of colony formation (bottom). **E** Secondary sphere formation capacity following knockdown or overexpression (OE) of NR5A2 for PDX215 and PDX354. In panels **C**, **D**, and **E** data are presented as mean ± SD and statistically analyzed using two-tailed Mann–Whitney tests to compare two groups (*n* = 4 biological replicates). Asterisks indicate significance at the indicated levels: * *p* < 0.05, *** *p* < 0.001, and **** *p* < 0.0001. Please also see Supplementary Fig. 3

We next evaluated the functional effects of NR5A2 inhibition on pancreatic CSCs. First, we treated forming spheres with Cpd3 every 48 h. Cpd3 treatment resulted in a marked inhibition of 3D sphere formation, significantly reducing the number and size of spheres compared to the control (Fig. 3A-B). To further corroborate these data, we treated PDAC cells during the formation of secondary spheres with Cpd3 for 72 h, which showed a sustained decrease in secondary sphere numbers, although this came with a degree of intertumoral variability (Fig. 3C). A consistent decrease in colony numbers on day 21 compared to the control was also noted (Figs. 3D & S3A). These data suggested that Cpd3 significantly reduced the number of CSCs with subsequent loss of sphere and colony-forming capacity. Even after treatment withdrawal, there was no evidence for recovery of the stemness phenotypes. Our findings were further validated using genetic tools for modulating *NR5A2* and its effect on secondary sphere formation. Briefly, once the primary sphere formation was finished on day 7, spheres that were larger than 40 μm were collected, dissociated to a single-cell suspension, and then re-cultured for an additional 7-day duration. This process is aimed at further increasing the concentration of cells that display stem-like properties. Intriguingly, knock-down resulted in a consistent decrease in second-generation sphere-forming capacity, whereas NR5A2 overexpression enhanced it (Fig. 3E). Consistent data were obtained for colony formation (Fig. S3B).

### Inhibition of *NR5A2* specifically eliminates tumor-initiating CSCs

**Fig. 4:**
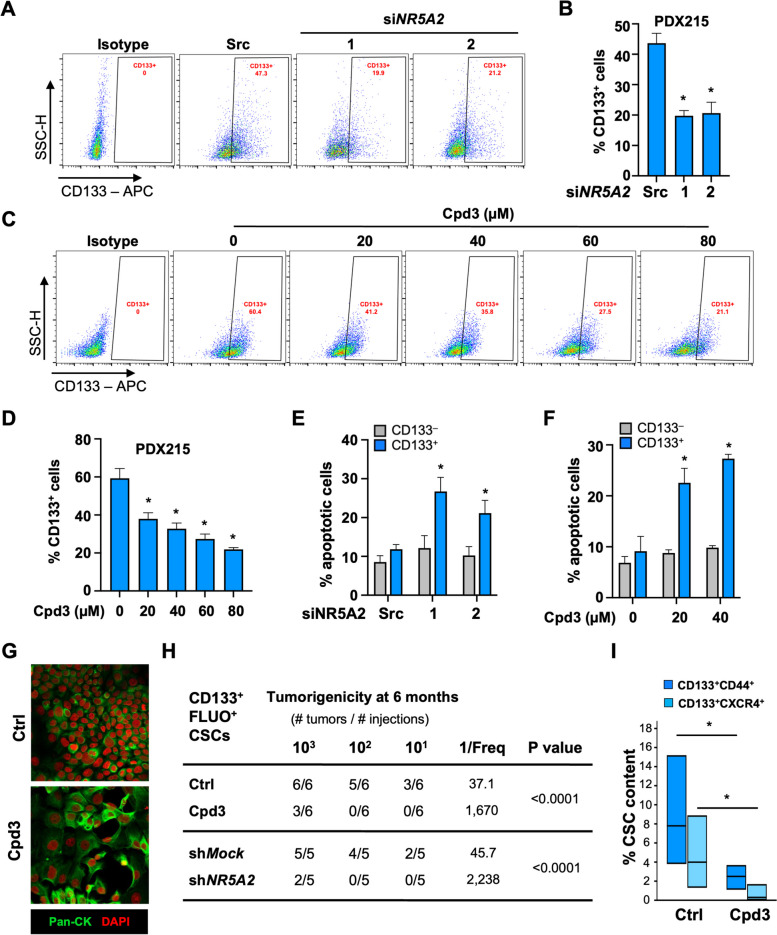
Inhibition of *NR5A2* specifically eliminates pancreatic cancer stem cells*.* **A** Flow cytometry analysis of CD133^+^ CSCs following 72 h of treatment with si*NR5A2* variants #1 and #2; 'src' indicates siScramble. Representative flow cytometry dot plots are displayed. **B** Quantification of *n* = 3 biological replicates. **C** Flow cytometry analysis of CD133^+^ CSCs following 72 h of treatment with graded doses of Cpd3. Representative data are depicted. **D** Quantification of CD133^+^ CSCs in *n* = 3 biological replicates. **E** Percentage of apoptotic Annexin V positive cells among CD133^–^ differentiated cancer cells (grey) and CD133^+^ CSC (blue) following treatment with si*NR5A2* variants #1 and #2, and **F** the percentage among CD133^–^ differentiated cancer cells (grey) and CD133^+^ CSC (blue) following treatment with 20 and 40 µM Cpd3 in *n* = 3 biological replicates. **G** Immunofluorescence for Pan-cytokeratin (green) following 48 h of treatment with 80 µM Cpd3. Nuclei were stained with DAPI (red). **H** In vivo tumorigenicity of decreasing numbers of highly enriched CD133^+^ FLUO^+^ CSCs following pharmacological or genetic targeting of *NR5A2*. **I** Flow cytometry for CD133^+^ CD44^+^ and CD133^+^ CXCR4^+^ CSCs in harvested tumors. In panels **A** to **F**, data are presented as mean ± SD and statistically analyzed using two-tailed Mann–Whitney tests to compare two groups (*n* = 3 biological replicates). In panel **I**, data are presented as floating bars, and statistically analyzed using two-tailed Mann–Whitney tests to compare two groups (*n* = 3–6 tumors). Asterisks indicate significance at the indicated levels: * *p* < 0.05. Please also see Supplementary Fig. 4

To further explore a potential bifunctional role of *NR5A2* in PDAC, we next investigated its specific role in the CSC subpopulation. For this purpose, we treated second-generation spheres that are highly enriched for CSCs with si*NR5A2* for 72 h and closely monitored their CD133^+^ CSC content. As depicted in Fig. 4A-B, treatment with si*NR5A2* resulted in a decrease in CD133^+^ CSCs. These findings prompted us to investigate whether a similar treatment effect could be observed when targeting *NR5A2* with graded doses of Cpd3. Intriguingly, we observed a dose-dependent reduction in the CD133^+^ CSC population following treatment with Cpd3 (20 to 80 µM) (Figs. 4C-D & S3C). Remarkably, this decline in the CD133^+^ CSC population was accompanied by a substantial increase in Annexin V staining, indicative of apoptosis specifically within the CD133^+^ CSC population. This effect could be reproduced by pharmacological inhibition of *NR5A2* using Cpd3 treatment and confirmed with si*NR5A2* (Figs. 4E-F & S3D-E). Importantly, the differentiated CD133^–^ cancer cells did not exhibit a significant increase in Annexin V staining (Fig. 4E-F), highlighting the specificity of the apoptotic response in the CD133^+^ CSC population. Notably, PDAC cells following treatment with Cpd3 showed a more pronounced epithelial-like morphology and increased cytokeratin expression (Figs. 4G & S3F). These data demonstrate that *NR5A2* has distinct roles in CSCs *versus* differentiated cancer cells. While *NR5A2* drives cell proliferation in differentiated cells, predominantly via downregulation of p21 (*CDKN1A*) [11], in CSCs, *NR5A2* appears to promote stemness and prevent apoptosis.

In vivo tumorigenicity represents the ultimate test for CSC functionality. Pancreatic CSCs can be enriched to the highest purity through their inherent autofluorescence (FLUO) and the stem cell marker CD133 [7, 9]. Therefore, we sorted CD133^+^FLUO^+^ CSCs and pharmacologically or genetically inhibited *NR5A2* prior to injecting them into immunocompromised mice. Tumor formation was monitored for six months after injection. As few as ten vehicle-treated CD133^+^FLUO^+^ CSCs readily formed tumors, whereas their *NR5A2*-inhibited counterparts failed to do so (Fig. 4H). Extreme limiting dilution analysis (ELDA) revealed a 45-fold and 49-fold decrease in CSC frequency upon pharmacological or genetic inhibition of *NR5A2*, respectively. The few tumors that did form after injection of larger numbers of Cpd3-treated cells showed a significant decrease in CSC content, defined as CD133^+^CD44^+^ or CD133^+^CXCR4^+^ cells (Fig. 4I). These data demonstrated that stemness in PDAC is dependent on *NR5A2*.

### *NR5A2* promotes stemness via direct binding to the SOX2 promoter/enhancer

**Fig. 5:**
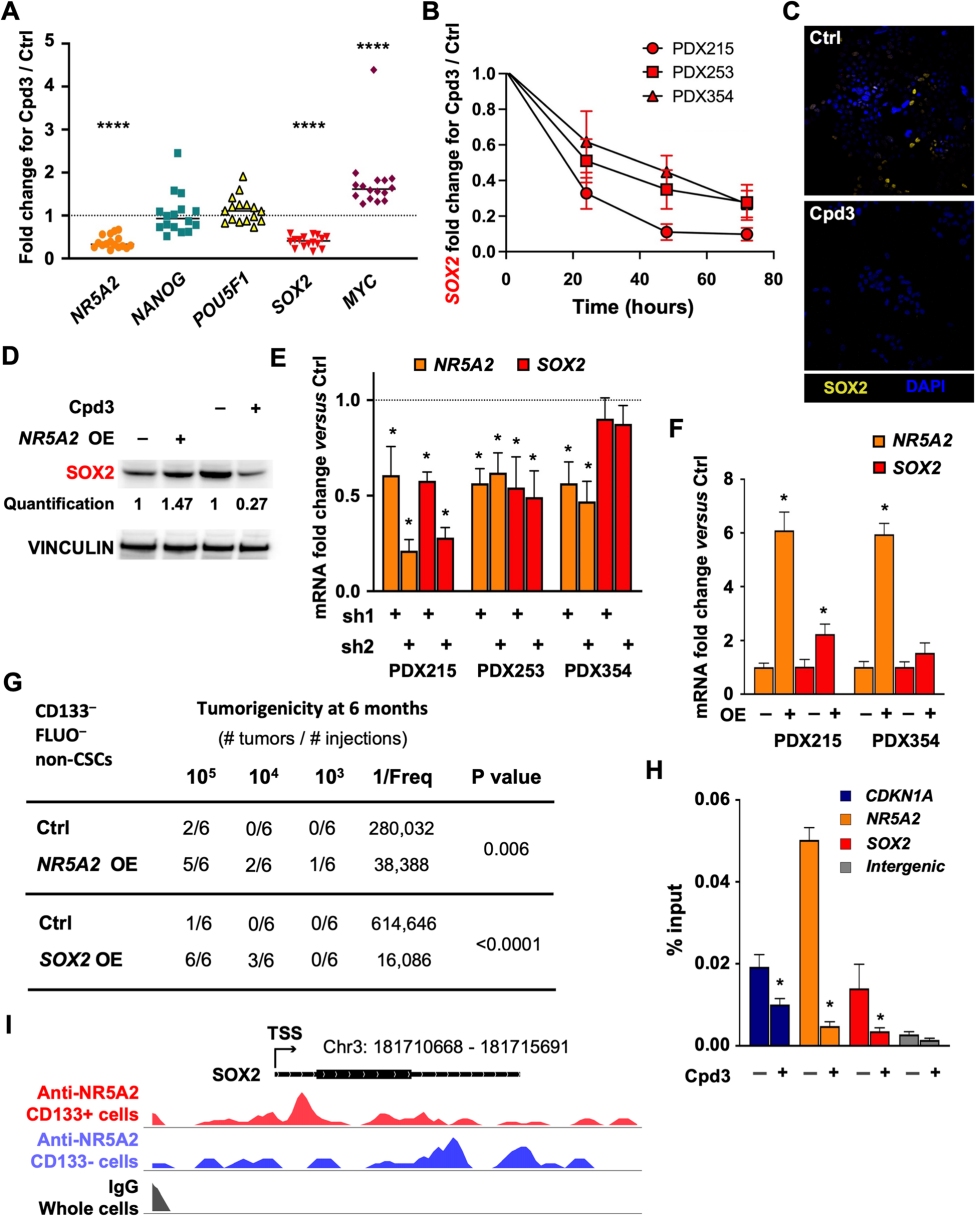
*NR5A2* promotes stemness via direct binding to the *SOX2* promoter/enhancer. **A** qPCR analysis for mRNA levels of stemness-associated genes following 24 h of treatment with Cpd3 (40 µM, *n* = 4 biological replicates with four technical replicates). The dotted line indicates baseline expression levels, set as 1.0. **B** Time course for *SOX2* RNA levels following Cpd3 treatment (40 µM) at 24, 48, and 72 h. **C** Immunofluorescence for SOX2 (yellow) following control (Ctrl) DMSO (top) or Cpd3 (bottom) treatment (40 µM) for 72 h. Nuclei are stained with DAPI (blue). **D** Western blot for SOX2 protein levels following *NR5A2* overexpression (left) or 72 h of treatment with Cpd3 (40 µM) (right). **E** qPCR analysis of mRNA levels for *NR5A2* and *SOX2* in response to knockdown of *NR5A2* using two different sh*NR5A2* (sh) in three different PDAC cultures. The dotted line indicates baseline expression levels, set as 1.0. **F** qPCR analysis of mRNA levels for *NR5A2* and *SOX2* in response to *NR5A2* overexpression (OE) in two different PDAC cultures. **G** In vivo tumorigenicity of decreasing numbers of the most differentiated CD133^–^FLUO^–^ pancreatic cancer cells following overexpression (OE) of *NR5A2* or *SOX2*. **H** Percent input of immunoprecipitated DNA at *CDKN1A* positive control enhancer, *NR5A2* and *SOX2* promoters. The intergenic region is used as a negative ChIP control. **I** CUT&Tag analysis of NR5A2 protein binding at the SOX2 locus. WashU Epigenome browser tracks showing CUT&TAG signals at the *SOX2* locus with the indicated transcription start site (TSS). Red signals represent NR5A2 binding in CD133^+^ PDAC cells (upper track), and purple signals represent NR5A2 binding in CD133^-^ PDAC cells (middle tracks). The black tracks represent the control, consisting of IgG binding in unsorted cells. In panels **B**, **E**, **F**, **G**, and **I**, data are presented as mean ± SD and statistically analyzed using two-tailed Mann–Whitney tests to compare two groups (*n* = 4 biological replicates). Asterisks indicate significance at the indicated levels: * *p* < 0.05 and **** *p* < 0.0001

To identify the mechanism by which *NR5A2* promotes stemness in PDAC, we screened for the expression of stemness-related transcription factors following treatment with Cpd3 [27, 28]. qPCR was performed after 24 h of *NR5A2* inhibition and revealed a significant and reproducible down-regulation of the *Sex-determining region Y-BOX-2* (*SOX2*) transcription factor and a rather unexpected up-regulation of *MYC*, whereas other stemness-related transcription factors such as *NANOG* and *POU5F1* (*OCT-4*) remained unchanged (Figs. 5A & S4A-B). These data suggest that *NR5A2* might control stemness in pancreatic CSCs by upregulating *SOX2* expression and repressing *MYC* expression through direct or indirect mechanisms.

The transcription factor *SOX2* is an oncogene strongly upregulated in CSC-enriched spheroid cultures (Fig. S4C). It is a functional driver of CSC phenotypes in PDAC [22, 29]. However, means to modulate *SOX2* levels have yet to be described. To determine if *SOX2* could be a direct target of *NR5A2,* we further explored the kinetics of *SOX2* downregulation following Cpd3 treatment in several PDAC models. Indeed, Cpd3 substantially decreased *SOX2* mRNA levels as early as 24 h, and levels continued to decrease to less than 50% within 72 h (Fig. 5B). Consistently, SOX2 protein levels were significantly reduced by 72 h, as shown by immunofluorescence (Figs. 5C & S4D) and western blot (Fig. 5D). In contrast, other transcription factors such as *NANOG*, *POU5F1,* and *KLF4* were not altered at 24 h (Fig. S4E). It was not until 72 h that we found their expression levels to be reduced. Therefore, most likely, they occurred secondary to the downregulation of *SOX2*.

We next used genetic targeting of *NR5A2* by lentiviral delivery of sh*NR5A2* for permanent suppression of *NR5A2*. We found a consistent decrease in *SOX2* mRNA (Fig. 5E), whereas lentiviral overexpression of *NR5A2* further upregulated *SOX2* mRNA (Fig. 5F) and protein levels (Fig. 5D). Intriguingly, using sorted differentiated CD133^–^FLUO^–^cancer cells, overexpression of *NR5A2* induced upregulation of CD133 and CXCR4 (Fig. S4F), enhanced sphere formation (Fig. S4G), and, most importantly, markedly enhanced in vivo tumorigenicity (Fig. 5G). Consistent data were obtained for the direct overexpression of *SOX2* (Figs. 5G & S4G).

The above data suggested that *NR5A2* promotes stemness in PDAC by directly controlling *SOX2* expression. To demonstrate such potential direct regulation of *SOX2* transcription by NR5A2, we performed a chromatin immunoprecipitation assay for NR5A2 at the promoter of *SOX2* and *NR5A2* (Fig. 5H). We found an enrichment of NR5A2 binding at both sites relative to the negative intergenic region, comparable to the published binding of NR5A2 at the *CDKN1A* (p21) enhancer [11]. Notably, this binding was virtually abrogated following treatment with Cpd3. These results demonstrate a direct regulation of *SOX2* expression by NR5A2, which can be abolished with Cpd3 treatment. Moreover, analysis of the CUT&TAG signals at the SOX2 locus revealed distinct binding patterns of NR5A2 in CD133^+^ versus CD133^–^ cells (Figs. 5I & S4H). In CD133^+^ CSCs, signals indicative of NR5A2 binding were observed directly after the TSS, consistent with NR5A2 binding to the SOX2 promoter region. In contrast, for CD133^–^ cells, signals indicative of NR5A2 binding appeared at a distant site downstream from the TSS. These data suggest that NR5A2 predominantly regulates SOX2 in CD133^+^ pancreatic cancer stem cells.

### *NR5A2* regulates CSC metabolism by modulating *MYC* expression

**Fig. 6:**
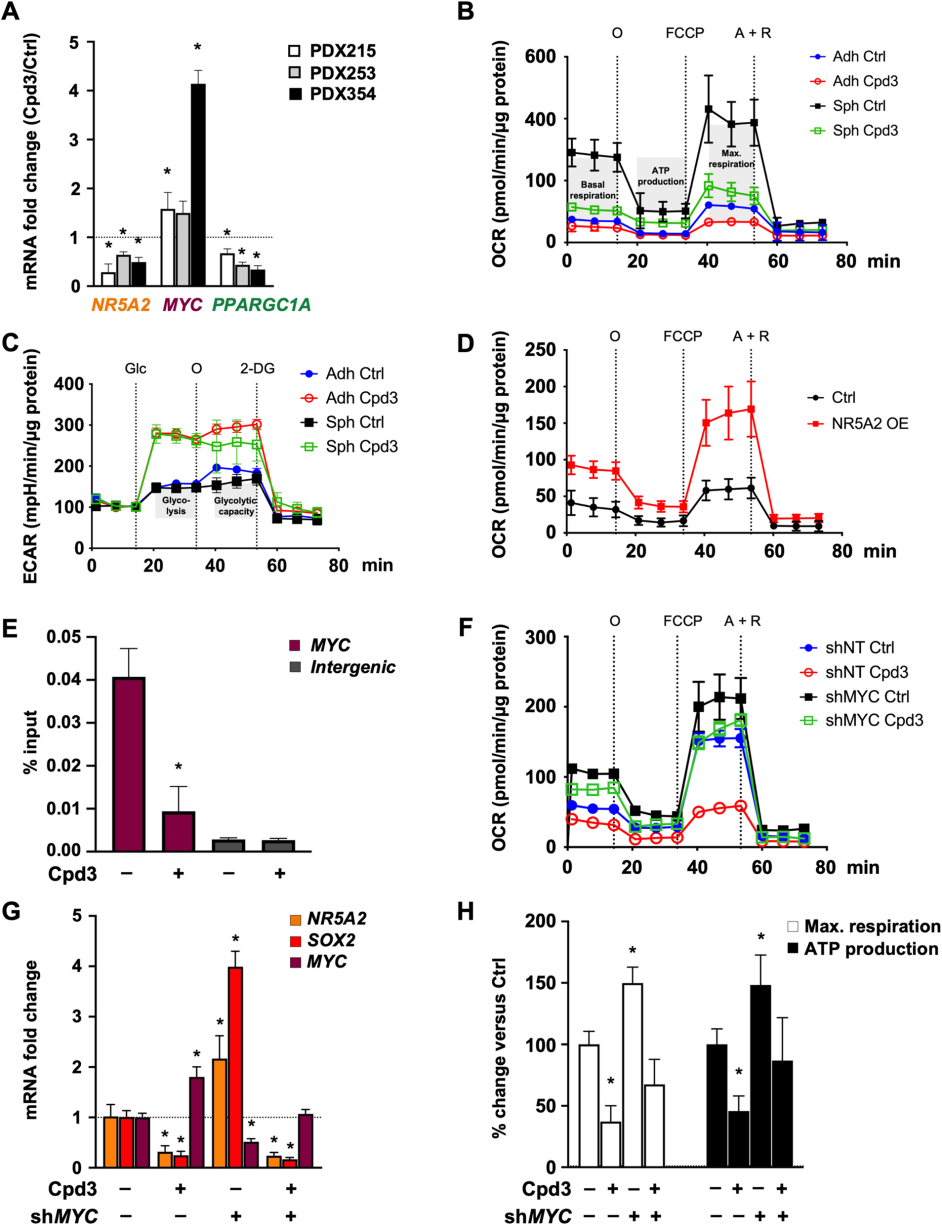
*NR5A2* promotes stemness by diminishing *MYC* expression*.* **A** qPCR analysis for *NR5A2*, *MYC* and *PPARGC1A* mRNA levels following 72 h of treatment with Cpd3 (40 µM). **B** Change in oxygen consumption rate (OCR) indicative of mitochondrial respiration following 72 h of treatment with Cpd3 (40 µM) for adherent cultures (Adh, differentiated cancer cells) or sphere cultures (Sph, enriched for CSCs). **C** Change in extracellular acidification rate (ECAR) indicative of glycolysis following 72 h of treatment with Cpd3 (40 µM) in Adh or Sph cultures. **D** Change in OCR following overexpression (OE) of *NR5A2* in differentiated cancer cells. **E** Percent input of immuno-precipitated DNA at the *MYC* promoter following treatment with Cpd3 (40 µM). The intergenic region is used as a negative ChIP control. **F** OCR levels for sh*NT* or sh*MYC*, following 72 h treatment with DMSO (Ctrl) or Cpd3 (40 µM). **G** qPCR of mRNA levels for *NR5A2*, *SOX2* and *MYC* in sh*NT* or sh*MYC* cells, following 72 h of treatment with DMSO (–) or Cpd3 (40 µM). **H** Changes in maximal (max.) respiration and ATP production in sh*NT* or sh*MYC* cells, following 72 h treatment with DMSO Ctrl (–) or Cpd3 (40 µM). In panels **A**, **E**, **G**, and **H** data are presented as mean ± SD and statistically analyzed using two-tailed Mann–Whitney tests to compare two groups (*n* = 4 biological replicates). The asterisk * indicates significance for *p* < 0.05. Please also see Supplementary Fig. 5

We have previously shown that an intricate balance of *MYC* and *PPARGC1A* (encoding for PGC-1α) determined the metabolic phenotype of pancreatic CSCs due to their strong reliance on oxidative phosphorylation for maintaining stemness [30]. High *MYC* expression suppresses *PPARGC1A*, mitochondrial biogenesis, and oxidative phosphorylation, thereby pushing metabolism towards glycolysis and diminishing stemness*.* Stimulated by the unexpected upregulation of *MYC* mRNA expression upon NR5A2 inhibition (Fig. 5A), we hypothesized that this might contribute, at least in part, to the loss of stemness. Therefore, we investigated the *MYC/PPARGC1A* balance following Cpd3 treatment and found that three different PDAC models showed increased *MYC* levels while *PPARGC1A* levels were down-regulated (Fig. 6A).

This inverse gene expression change resulted in a drastic switch of the metabolic phenotype of pancreatic CSCs from their preferred oxidative phosphorylation state to a highly glycolytic phenotype (Fig. 6B-C). This switch was associated with a reduced dependency of CSC-enriched spheres on mitochondrial respiration, maximal respiration, and ATP production (Fig. S5A). On the other hand, *NR5A2* overexpression in differentiated cancer cells shifted their metabolism toward oxidative phosphorylation (Fig. 6D). To conclusively demonstrate a potential direct regulation of *MYC* transcription by NR5A2, we performed a chromatin immunoprecipitation assay for NR5A2 at the *MYC* enhancer. We found an enrichment of NR5A2 binding to the *MYC* enhancer relative to the negative intergenic region (Fig. 6E). Importantly, treatment with Cpd3 virtually abrogated this binding. These results demonstrate a direct regulation of *MYC* expression by NR5A2, which can be abolished with Cpd3 treatment.

To determine whether the cellular metabolic phenotype induced by NR5A2 inhibition was indeed functionally mediated by unleashed *MYC* expression, we targeted *MYC* expression levels using an inducible shRNA against *MYC*. As predicted, in untreated PDAC cells, *MYC* knock-down increased mitochondrial respiration (Fig. 6F), reduced lactate production (Fig. S5B), and subsequently enhanced CSC function, as evidenced by augmented sphere formation capacity (Fig. S5D). These metabolic and phenotypic changes coincided with the upregulation of *SOX2* and *NR5A2* (Fig. 6G). Intriguingly, the simultaneous knock-down of *MYC* virtually abrogated the expected metabolic shift toward enhanced glycolysis and glycolytic capacity in response to Cpd3 treatment. Consistently, *MYC* knock-down also attenuated the increase in lactate production and reversed the impaired sphere formation capacity following Cpd3 treatment (Figs. 6F-H & S5B-D). These data demonstrate that, besides *SOX2* activation, inhibition of *MYC* is an essential mechanism for the stemness-promoting effects of NR5A2, resulting in metabolic reprogramming of the cells toward oxidative phosphorylation.

### *NR5A2 *inhibition targets CSC in vivo and prevents disease relapse

**Fig. 7:**
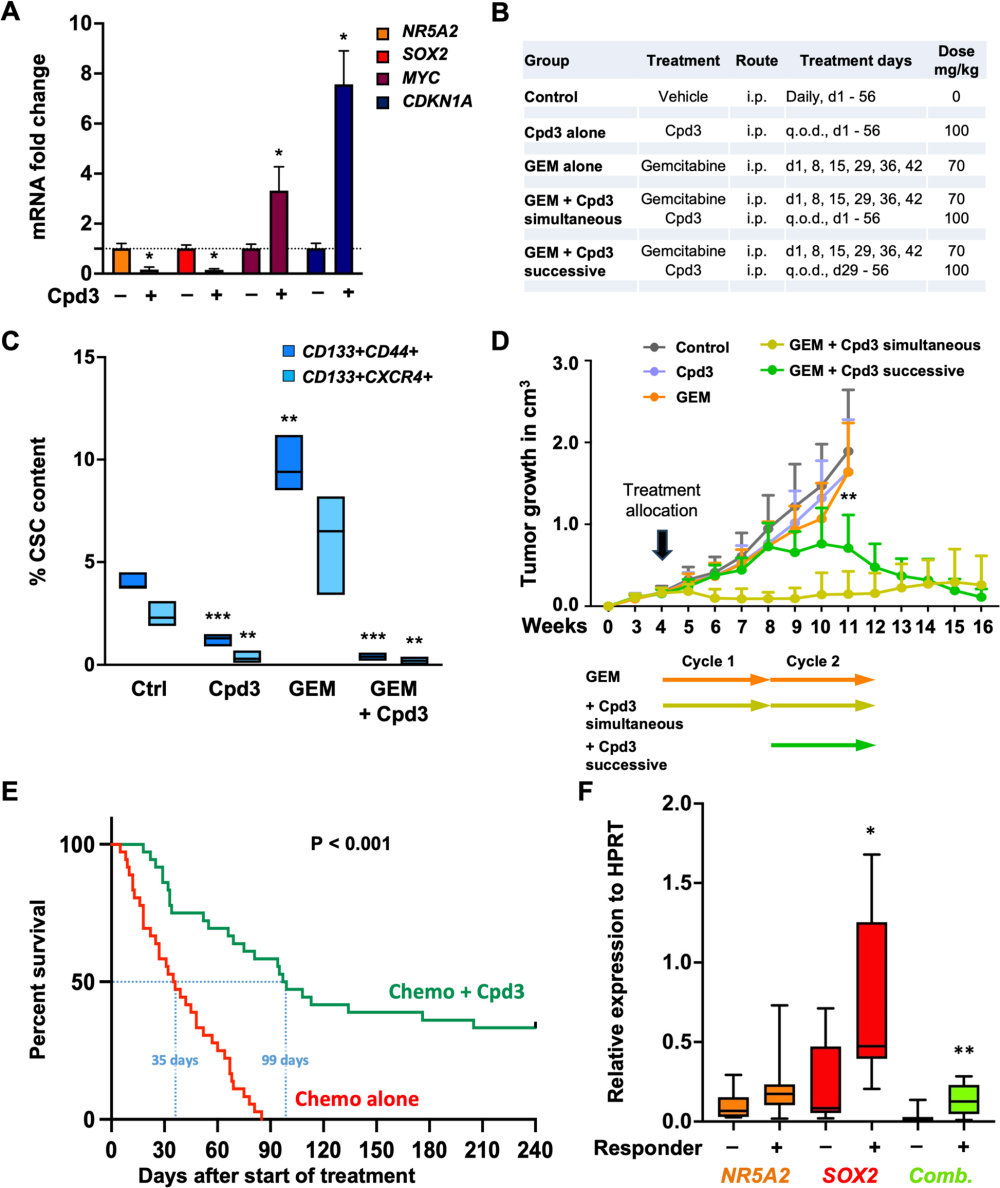
NR5A2 inhibition targets CSCs in vivo and extends survival in preclinical PDAC models. **A** qPCR analysis for *NR5A2*, *SOX2*, *MYC* and *CDKN1A* mRNA levels following 72 h of treatment with Cpd3 (100 mg/kg body weight) in vivo. Data are presented as mean ± SD (*n* = 4 biological replicates). **B** Dosing and timing of allocated treatments for tumor-bearing mice. **C** CSC content following seven days of allocated treatments. CSCs were defined as CD133^+^ CD44^+^ cells or CD133^+^ CXCR4^+^ cells as assessed by flow cytometry. Data are presented as floating bars with lines indicating the median for *n* = 3 tumors per group and statistically analyzed using two-tailed t-tests to compare versus control. **D** Tumor growth in cm^3^ according to allocated treatments with two treatment cycles of 28 days each as outlined in (**B**), with *n* = 8–9 mice per group. Each mouse carried two tumors. Data are presented as mean ± SD. The arrows below depict treatment cycles, rather than specific treatment intervals or durations within each cycle. **E** Overall survival of tumor-bearing mice following allocated treatment. A total of 18 PDX models were treated using a 2 × 1 × 1 approach (two animals per model per treatment); *n* = 36 per group. **F** qPCR analysis of baseline *NR5A2* and *SOX2* mRNA levels (*n* = 8–10 biological replicates). Combined gene expression represents the mathematical product of *NR5A2* and *SOX2* mRNA levels. Data are presented as box and whisker plots with the center line denoting the median value. In panels **A**, **D**, and **F** data are statistically analyzed using two-tailed Mann–Whitney tests to compare two groups. Asterisks indicate significance at the indicated levels: * *p* < 0.05, ** *p* < 0.01, and *** *p* < 0.001. Please also see Supplementary Fig. 6

The above data suggest that NR5A2 inhibition could be used as a therapeutic strategy to counteract stemness in PDAC. To demonstrate our findings' potential clinical utility, we performed in vivo intervention studies. In pilot experiments, we used our PDX354 model as an intermediate responder to Cpd3. Due to the lipophilic characteristics of Cpd3, we encapsulated the compound into lipid nanocarriers before i.p. injection. We validated the biological activity of encapsulated Cpd3 (100 mg/kg body weight) by qPCR analysis, showing strong downregulation of *NR5A2* and *SOX2* with a consistent upregulation of *MYC* and *CDKN1A* in vivo (Fig. 7A).

Next, once PDX354 tumors had reached ~ 0.2cm^3^ in volume, mice were randomized to vehicle control, Cpd3 alone, gemcitabine alone, or gemcitabine plus Cpd3. The latter was either given early and concomitantly with gemcitabine (during the first and second cycles of chemotherapy) or delayed (only during the second cycle of chemotherapy) (Fig. 7B). We assessed CSC content at the end of the first treatment cycle (day 28). Cpd3 alone significantly reduced the CSC content defined as CD133^+^CD44^+^ or CD133^+^CXCR4^+^ cancer cells, whereas gemcitabine treatment increased the CSC content (Fig. 7C). The combined simultaneous treatment with gemcitabine and Cpd3 virtually reduced all CSCs to undetectable levels in the tumors.

Notably, the reduced CSC content induced by Cpd3 monotherapy did not translate into significantly reduced tumor growth (Fig. 7D). However, the simultaneous combination of Cpd3 and gemcitabine resulted in instant tumor regression, with no overt relapse over the subsequent 12 weeks. We also tested a successive treatment strategy for gemcitabine and Cpd3 to reduce potential side effects and to avoid the antiproliferative effect of Cpd3, at least during the first cycle of chemotherapy. The successive treatment with delayed Cpd3 treatment still resulted in significant and sustained tumor regression, despite the much larger tumor burden at the time of Cpd3 treatment initiation (Fig. 7D). Notably, Cpd3 treatment initiation at this advanced disease state was associated with a significant but reversible drop in body weight (Fig. S6).

As our in vitro data suggested heterogeneity in response to Cpd3 treatment as evidenced by varying degrees of target inhibition (Fig. 5), we wanted to assess the share of responsive PDAC phenotypes in vivo. For this purpose, we compiled a panel of 18 randomly selected PDAC models (listed in Materials & Methods). We applied a 2 × 1 × 1 study design (two animals per model per treatment) [31] with overall survival during the 35-week follow-up as the primary endpoint. PDAC models were treated with either chemotherapy (Chemo: gemcitabine + nab-paclitaxel) alone or Chemo plus Cpd3 (concomitantly, 100 mg/kg body weight). Overall median survival was tripled with 99 days for Chemo plus Cpd3 compared to 35 days for Chemo only (Fig. 7E). More importantly, 33% of the PDAC models treated with Chemo plus Cpd3 survived the entire follow-up period of 240 days. To distinguish responders from non-responders, we correlated baseline mRNA levels for *NR5A2* and *SOX2* with response to treatment. Intriguingly, while *NR5A2* expression levels alone did not clearly separate responders from non-responders, its combination with *SOX2* levels showed a highly significant separation of the two groups (Fig. 7F).

## Discussion

Our findings establish the crucial role of NR5A2 as a regulator of stemness in pancreatic cancer. In the realm of PDAC stem cells, NR5A2 plays a dual role, acting to enhance the stemness-associated transcription factor, SOX2, while simultaneously suppressing the metabolic transcription factor, MYC. By leveraging both pharmacological and genetic tools to manipulate NR5A2 expression, we achieved the capacity to either inhibit stemness by impeding NR5A2 or stimulate stemness through the overexpression of NR5A2. In a comprehensive preclinical investigation featuring a diverse array of PDX models, we observed that inhibiting NR5A2 substantially extended the survival of a notable portion of aggressive PDAC models. Our tumor profiling results underline the importance of customizing treatment to tumors that exhibit the highest combined expression of NR5A2 and SOX2, thereby maximizing the response to therapy.

Previous research suggested an upregulation of NR5A2 in human pancreatic cancer cells compared to non-transformed cells [16, 32]. However, these investigations primarily involved immortalized pancreatic cancer cell lines, using non-neoplastic pancreatic ductal epithelial cells as controls. In contrast, our single-cell RNA-seq analysis of freshly resected PDAC tissue reveals that NR5A2 is downregulated in PDAC cells when compared to non-transformed ductal and acinar cells (Fig. 1F). Consistently, similar findings were confirmed by analyzing bulk tumor tissue in comparison to normal pancreas (Fig. 1D). These findings cast some doubt on the previous studies' conclusions [16, 32] due to potential technical limitations. Nonetheless, experiments employing immortalized pancreatic cancer cell lines demonstrated that inhibiting NR5A2 significantly suppressed cell proliferation, indicating its role in tumor growth [16]. Mechanistically, NR5A2 was found to inhibit p21 expression (encoded by the CDKN1A gene) [11, 33]. Recent studies have also suggested that NR5A2 may drive epithelial-to-mesenchymal transition (EMT) and stemness, even though they lacked mechanistic links or robust in vivo evidence [32]. In line with this notion, a genome-wide association study identified multiple pancreatic cancer susceptibility loci, including several SNPs located within a region on chromosome 1q32.1 where NR5A2 resides [34]. Furthermore, the recently introduced ADEX class for PDAC is characterized by transcriptional networks crucial during pancreatic development and differentiation. This class represents a subset of pancreatic progenitor tumors, with key networks showing upregulation of transcription factors like NR5A2, MIST1, and RBPJL, along with their downstream targets [34].

Mouse studies conducted in the context of the pancreas have provided valuable insights into the pivotal role of Nr5a2. This transcription factor emerges as a key player in maintaining acinar homeostasis, safeguarding the identity of acinar cells and facilitating their restoration during regeneration. Consequently, it acts as a limiting factor in the development of pancreatic carcinogenesis induced by oncogenic Kras [17]. Consistently, under conditions of genetic constraint, Nr5a2 undergoes a significant transcriptional shift. It transitions from a specialization in acinar differentiation to promoting the activation of inflammatory genes, fostering a basal pre-inflammatory state within the pancreas [35]. Further evidence highlighting the potential implications of Nr5a2 in carcinogenesis is found in studies involving the intestine. Here, Nr5a2 collaborates with β-catenin to stimulate the expression of cell cycle genes. Intriguingly, when *Nr5a2* is haploinsufficient, it results in a reduction of intestinal tumor formation [36]. Collectively, these mouse studies affirm that Nr5a2 serves as a guardian of acinar differentiation in the adult murine pancreas, thwarting the onset of pancreatic carcinogenesis. However, under certain circumstances, it can shift its role towards promoting inflammation and potentially acting as an instigator of carcinogenesis.

Our study further dissects the distinct cellular effects of *NR5A2* across specific PDAC phenotypes using the most clinically relevant PDAC models and shows that the function of *NR5A2* in PDAC cells is highly cell context dependent. In differentiated cancer cells, we validated the previously reported p21-mediated antiproliferative effect of *NR5A2* inhibition (Fig. 2). However, in the smaller subset of pancreatic CSCs, *NR5A2* has distinct functions and controls stemness phenotypes. Inhibition of *NR5A2* using genetic or pharmacological tools resulted in a loss of stemness, and subsequent differentiation and apoptosis (Fig. 4). These alterations translated into a virtually complete abrogation of in vivo tumorigenicity. In contrast, overexpression of *NR5A2* induced in vivo tumorigenicity of most differentiated cancer cells (Fig. 5H).

Mechanistically, we show for the first time that *NR5A2* promotes stemness in pancreatic cancer cells via enhanced expression of *SOX2* (Sex-determining region Y (SRY)-Box2). *SOX2* is a well-characterized pluripotency factor essential for stem cell self-renewal, reprogramming, and homeostasis [37]. The cellular levels of *SOX2* are precisely regulated by a complicated network at the transcription, post-transcription, and post-translation levels. *SOX2* expression is undetectable in normal pancreatic acinar or ductal cells, but the ectopic expression of *SOX2* has been reported for a large fraction of human pancreatic tumors and was particularly enriched in CSCs [29]. *SOX2* overexpression in PDAC and other cancer types promotes cancer cell invasion/metastasis, stemness, and drug resistance and is commonly associated with poor survival [38]. While overexpression of *SOX2* is frequently related to gene amplification, it has not been reported for PDAC [39]. Notably, *SOX2* expression in PDAC is highly heterogeneous and mostly restricted to the CSC compartment (Fig. S4C) [29]. Therefore, an epigenetic mechanism for *SOX2* overexpression in PDAC seemed more likely. Indeed, our data now demonstrate that *SOX2* expression in pancreatic CSCs is regulated by the upstream transcriptional mediator *NR5A2* via direct binding to its promoter/enhancer.

Efforts to develop selective SOX2 inhibitors have been limited, primarily due to SOX2's undruggable nature and its critical role in normal adult stem cells [40]. Our research highlights the potential of targeting NR5A2, which binds to the SOX2 promoter/enhancer and drives its expression. NR5A2 is mainly expressed in the pancreas and liver, making it an attractive target for selectively inhibiting SOX2 in pancreatic ductal adenocarcinoma (PDAC). Inhibiting NR5A2 resulted in a rapid downregulation of SOX2, while other stemness-related factors were minimally affected, suggesting a selective impact on SOX2 (Fig. 5E), which could then be validated by ChIP (Fig. 5I). Further studies revealed that NR5A2 also played a role in suppressing MYC, an inhibitor of oxidative phosphorylation, which is crucial for maintaining stemness in PDAC [30]. While differentiated PDAC cells are indeed running on glycolysis, as originally proposed by Warburg [41], the CSC compartment in PDAC relies on oxidative phosphorylation for maintaining stemness phenotypes [30, 42]. Inhibition of NR5A2 led to a metabolic shift in cancer stem cells towards glycolysis, reducing their stemness features.

Combining a highly specific NR5A2 inhibitor, Cpd3, with standard chemotherapy showed promising results. This combination led to a synergistic effect, resulting in tumor regression and extended survival in PDAC models. Notably, simultaneous use of Cpd3 and chemotherapy was more effective than a staggered approach, although it came with some increased toxicity. In a broader study involving patient-derived xenograft models, the combination of Cpd3 and chemotherapy tripled median survival and induced remission in a third of the models (Fig. 7E). Notably, those with simultaneous expression of both NR5A2 and SOX2 were more likely to respond (Fig. 7F). Of note, Cpd3 monotherapy had limited benefits, emphasizing the importance of combining NR5A2 inhibition with chemotherapy to target both cancer stem cells and the larger population of differentiated PDAC cells. Further investigations are needed to explore combinatorial approaches, effects on the tumor microenvironment, and the immune system's influence on this therapeutic approach.

In conclusion, NR5A2 shows promise as a target for PDAC therapy, but its combination with chemotherapy is essential for optimal outcomes. Tailoring treatments based on patient phenotypes could further improve response rates. The potential of circulating tumor cells as non-invasive biomarkers for assessing NR5A2 and SOX2 expression should be explored. It is important to note that our data are from preclinical studies, and further research and clinical trials are necessary to confirm the efficacy and safety of this approach, potentially improving PDAC patient outcomes.

## Experimental procedures

### Culture and treatment of primary human PDAC cells

PDAC patient samples were collected and propagated at the Spanish National Cancer Research Centre (CNIO), Madrid, Spain (reference 1204090835CHMH), the ARC-Net Biobank of the University and Hospital Trust of Verona approved by the Verona University Hospital Ethics Committee (Program 1885 protocol 52,438 23/11/2010, program 2172 protocol 26,773 23/05/2012), and the Jiao Tong University School of Medicine (reference 2013–0905-70), respectively. PDX tissues, grown subcutaneously in the flank of immunodeficient BALB/c nude mice (Charles River), were used to isolate primary PDAC cells with 2% collagenase P (Roche, UK) and 1 mg/mL dispase (Life Technologies, UK). Minced fragments of PDX-derived tumor tissues were digested with Collagenase (Stem Cell technologies for 1.5 h at 37 °C as previously described (Mueller et al., 2009). Cells were cultured in RPMI media supplemented with 10% FBS and 50 units/ml penicillin/streptomycin. A final concentration of 10 nM of siRNA targeting the *NR5A2* gene (QIAGEN- 1,027,416) and negative control (QIAGEN-1027281) were used to transfect the primary PDAC cells with HiPerFect transfection reagent (QIAGEN-301705). The siRNA sequences are provided in Supplementary Table 3. The NR5A2 inhibitor “Cpd3” (Sigma-Aldrich-505601) was dissolved in DMSO at 10 mM stock concentration and applied to the desired final concentrations in cell cultures.

### CSC-enriching sphere cultures

Primary cell suspensions were cultured for seven days in ultra-low attachment plates (Corning) at 10,000 cells/mL density in DMEM-F12 (Invitrogen) supplemented with B-27 (GIBCO) and bFGF (PeproTech EC). For serial passaging, termed 2^nd^ generation sphere formation, 7-day spheres were retained using a 40 µm cell strainer, dissociated into single cells with trypsin, and reseeded at 10,000 cells/ml in the same conditions for another seven days. The number of primary and secondary spheres was determined using a CASY cell counter (Roche).

### Colony formation assay

Cells were treated with Cpd3 when adherent, dissociated by trypsin, and seeded at low density (500 or 1,000 cells) in a 6-well plate. Colonies are left to form for 21 days, then fixed and stained with Crystal violet. Crystal violet was dissolved in 1% SDS and absorbance read at 570 nm using Fluostar optima plate reader (BMG Labtech).

### Cell toxicity/viability assays

Cells were seeded at 5,000 cells per well in a 96-well format and treated the following day with desired concentrations of Cpd3 for 24 and 72 h, respectively. Media was recovered for adenylate kinase (AK) release using ToxiLight Non-destructive Cytotoxicity BioAssay Kit according to the manufacturer’s instructions (Lonza). For cell viability, cells were analyzed using the CellTiter-Glo® Luminescent Cell Viability Assay (Promega) according to the manufacturer’s instructions.

### Incucyte™ platform

The label-free live-cell imaging system (Sartorius) was used to assess cell expansion alone or in combination with Incucyte™ caspase 3/7 dyes staining. The latter non-fluorescent dyes were used according to manufacturer’s instructions and detect apoptosis in real-time by binding activated caspase 3/7 to release a DNA-binding fluorescent label.

### Flow cytometry and cell sorting

Primary PDAC cells from adherent or spheres cultures were dissociated using trypsin and suspended in sorting buffer (PBS containing 3% FBS and 3 mM EDTA) and either sorted on FACS ARIA II (BD biosciences) or stained with antibodies anti-CD133/1-APC or PE (Miltenyi 130-090-826), anti-CXCR4-APC (Biolegend 306,510), CD44-PE (Biolegend 324,206), and appropriate isotype-matched control antibodies. DAPI was used for the exclusion of dead cells (eBiosciences). For Annexin V staining, cells were stained with Annexin V (550,474 eBiosicences) diluted in Annexin V binding buffer (556,454 eBiosciences) according to the manufacturer’s instructions. Ki-67 staining (BD biosciences 556,026) was used for proliferation scoring following manufacturer’s recommendations. Samples were processed using LSR Fortessa 2 and analyzed using FlowJo V10 software.

### In vivo tumorigenicity assay

Treated sphere cultures were dissociated with trypsin, suspended in 50 μl Matrigel™ (BD), then implanted subcutaneously into female 6–8 weeks old NU-*Foxn1*^*nu*^ nude mice (Harlan Laboratories) or BALB/c nude mice (Charles River). Tumor growth was monitored for up to 6 months after implantation. Procedures were conducted in accordance with the animals in science regulations (UK Project License PPL70/8129 and Shanghai Jiao Tong University Project Approval A-2020–004). Tumors were digested and stained to detect CSC content using flow cytometry.

### In vivo PDAC models

PDAC cells (1 × 10^5^ in 50 μl Matrigel™) were injected subcutaneously (Fig. 7D) or tumor pieces (Panc12558, 12560, 12650, 12706, 12708, 12708, 12709, 12911, 12912, 12975, 12976, 14863, 14836, 14837, 14838, 14839, 14840, 14841; Experimental Pharmacology and Oncology Berlin-Buch GmbH, Berlin, Germany) were implanted orthotopically (Fig. 7E) into 6–8-week-old BALB/c nude mice (Charles River) as described previously [7, 22, 43]. Once PDAC tumors had reached ~ 0.2 cm^3^, mice were randomly assigned to treatments as outlined in Fig. 7B. All animal procedures were conducted in accordance with the 3Rs and the animals in science regulations (Shanghai Jiao Tong University Project Approval A-2020–004).

### Preparation of Cpd3-loaded nanodrug delivery systems

Cpd3 was incorporated using the thin film hydration method [44]. Briefly, a mixture of soybean phosphatidylcholine, cholesterol, and Cpd3 at a mass ratio of 10:1:1 was dissolved in a mixed solvent of chloroform and methanol (2:1, v/v). After solvent removal by rotary evaporation, dried lipid films were hydrated in phosphate-buffered saline at 37 °C at a phospholipid concentration of 2.5 mg/mL. Following sonication at 37 °C for 15 min, Cpd3-loaded lipid-based nanocarriers were obtained. The non-encapsulated Cpd3 was removed by centrifugation (1,000 rpm, 10 min).

### Real-time PCR analysis

Total RNA was isolated using TRIzol reagent (Invitrogen) according to manufacturer, and cDNA synthesis was performed using Superscript Vilo reverse transcriptase (Invitrogen). A total of 10 ng of cDNA was used for real-time quantification. Primer sequences were provided in Supplementary Table 1. Samples were run on a QuantStudio 7 Real-Time PCR System (Applied Biosystems).

### RNA-seq library construction and analysis

Total RNA was isolated by the guanidine thiocyanate method using standard protocols [45]. RNA Integrity Numbers were in the range of 9.2 to 10.0 when assayed on an Agilent 2100 Bioanalyzer. PolyA^+^ RNA fraction was extracted and randomly fragmented, converted to double-stranded cDNA, and processed through subsequent enzymatic treatments of end-repair, dA-tailing, and ligation to adapters as in Illumina's "TruSeq RNA Sample Preparation v2 Protocol" (Part # 15,026,494 Rev. C). Adapter-ligated library was completed by eight cycles of PCR with Illumina PE primers. The resulting purified cDNA library was applied to an Illumina flow cell for cluster generation (TruSeq cluster generation kit v5) and sequenced on the Genome Analyzer IIx with SBS TruSeq v5 reagents by following the manufacturer's protocols. The 40-nt single-end RNA-seq sequenced reads were aligned to the human genome (GRCh37/hg19) with TopHat-2.0.4 [46] using Bowtie 0.12.7 [47] and Samtools 0.1.16 [48], allowing two mismatches and five multi-hits. Transcripts assembly and estimation of their abundances were calculated with Cufflinks 1.3.0 [46], using the human genome annotation data set Homo_sapiens.GRCh37.65 from Ensembl [49]. Differential expression for genes across the different conditions was calculated with Cuffdiff [46].

### Western blot analysis

Cell pellets were lysed in RIPA buffer (Sigma) supplemented with protease inhibitors (Roche). Total protein (30ug) was resolved on 4–12% Bis–Tris protein gels (Invitrogen) and transferred to nitrocellulose membranes (GE Healthcare). Membranes were blocked in PBS-tween 5%BSA for one hour at RT, followed by an overnight primary antibody incubation at 4 °C. We used the following primary antibodies: anti-NR5A2 (Abcam ab125034, dilution 1:2,000), anti-SOX2 (Cell signalling 3579, dilution 1:1,000), and anti-Vinculin (Sigma V9131, dilution 1:5,000). Secondary antibodies (DAKO, dilution 1:5,000) were incubated at room temperature for one hour, and blots were developed using Chemidoc Amersham Imager 600. Image J was used for band quantification.

### Immunofluorescence

Cells were fixed in 4% paraformaldehyde for 10 min at RT, followed by permeabilization with 0.5% Triton for 10 min at RT. Blocking was performed with 10% goat serum for two hours at RT. Incubation with anti-SOX2 (Cell Signaling Technology #3579) was conducted at 1:400 dilution at 4 °C overnight. For cytokeratin staining, cells were permeabilized with 0.2% Triton X-100 diluted in PBS containing 0.5% BSA and 2 mM EDTA for 10 min, followed incubation with FITC-conjugated anti-cytokeratin (CK3-6H5, Miltenyi Biotec) at 1:10 for 30 min. Nuclei were visualized by incubating the cells with DAPI for 5 min. Samples were mounted in Dako Faramount Aqueous Mounting Medium and images were captured using a fluorescent microscope (Ariol system, Genetix).

### Chromatin immunoprecipitation assay

ChIP was performed as previously described (Bracken et al., 2003). Briefly, cells were fixed in 1% formaldehyde and lysed in an SDS lysis buffer. The chromatin was sonicated to obtain fragments of ~ 500 bp, and ChIP was performed using 1 mg of protein lysate. This step was followed by incubation with 10 μg of anti-NR5A2 (R&D Systems PP-H2325-00) at 4 °C overnight. DNA was then eluted, reverse cross-linked, and extracted on Qiagen PCR extraction columns prior to quantitative PCR analysis. Primer sequences are provided in Supplementary Table 2.

### CUT&Tag (cleavage under targets and tagmentation)

CUT&Tag was performed on sorted PDAC cells, with CD133^+^ and CD133^-^ populations obtained through cell sorting. Unsorted cells served as the control. The procedure involved the permeabilization of cells and subsequent immobilization on concanavalin A-coated magnetic beads to aid in the washing steps. Cells were then incubated with a negative control IgG (SouthernBiotech 0107–01) or a primary antibody specific to the NR5A2 protein (R&D Systems PP-H2325-00), followed by incubation with a secondary antibody (Abcam ab6708). This was followed by the incubation with assembled transposomes, which consisted of protein A fused to the Tn5 transposase enzyme conjugated to NGS adapters. After stringent washing to remove unbound transposomes, the reaction was activated by the addition of Mg2^+^. This led to chromatin cleavage near the protein binding site and simultaneous addition of NGS adapter DNA sequences, facilitating chromatin cleavage and library preparation in a single step. All steps were performed according to the manufacturer's instructions.

### XF extracellular flux analysis

Single-cell suspensions from dissociated secondary spheres or adherent cultures were plated in XF96 Cell Culture Microplates (Seahorse Bioscience) pre-coated with Cell-Tak (BD Biosciences) at a density of 30,000 cells/well. For OCR determination, cells were incubated for 1 h in base assay medium (D5030, Sigma; supplemented with 2 mM glutamine, 10 mM glucose, and 1 mM pyruvate) prior to analysis using the XF Cell Mito Stress Kit (Seahorse Bioscience). Concentrations of oligomycin and FCCP were adjusted for each primary cell type [30]. For the analysis of glycolytic metabolism, cells were incubated in basal media prior to injections using the Glycolytic Test kit (Seahorse Bioscience). Experiments were run on the XF96 analyzer (Seahorse Bioscience), and raw data were normalized to protein content.

### Lactate production

Following treatments, according to the manufacturer’s instructions, lactate production in cell culture media was measured using the Lactate Assay Kit II (Sigma, St. Louis, MO).

### Lentiviral constructs

Utilized shRNA sequences against *NR5A2* are provided in Supplementary Table 3. For *MYC* knockdown, the shERWOOD UltramiR Lentiviral Inducible pZIP target gene set for *MYC* (TLHSU2300-4609) was used (Transomic Technologies, Huntsville, AL) [30]. *NR5A2* overexpression vector was also purchased from Transomic Technologies (TOLH-1518069). Third-generation lentiviruses were generated in 293T cells with the respective lentiviral backbone, PAX2 packaging plasmid, and VSV-G using Lipofectamine 2000 transfection reagent (Invitrogen).

### Statistical analyses

Results are represented as means ± SD unless stated otherwise. Comparison and statistical differences were calculated using two-tailed Mann–Whitney U tests, with significance considered at *p* values of * *p* < 0.05, ** *p* < 0.01, *** *p* < 0.001, and **** *p* < 0.0001. Additionally, survival analysis was performed using the log-rank test. All analyses were performed using GraphPad Prism 9.0 or SPSS v26 software.

### Supplementary Information

**Additional file 1: Supplementary Table 1.** cDNA primer sequences. **Supplementary Table 2.** Genomic DNA primer sequences. **Supplementary Table 3.** Genetic targeting of NR5A2.

**Additional file 2: Figure S1.** NR5A2 is overexpressed in pancreatic cancer stem cells. **Figure S2.** NR5A2 regulates proliferation of differentiated PDAC cells. **Figure S3.** NR5A2 controls stemness in PDAC. **Figure S4.** Inhibition of NR5A2 specifically eliminates tumor-initiating CSCs. **Figure S5.** NR5A2 promotes stemness by diminishing MYC expression. **Figure S6.** NR5A2 inhibition targets CSCs in vivo and extends survival in preclinical PDAC models.

## Data Availability

The data and material generated and analyzed during this study are included in the published article and its supplementary information files. Access to the data and material generated and analyzed during this study can be requested from the corresponding authors, who will consider all reasonable requests.
