# Supplementary material for: Inhibiting NR5A2 targets stemness in pancreatic cancer by disrupting SOX2/MYC signaling and restoring chemosensitivity
Source: J Exp Clin Cancer Res. 2023 Nov 28;42:323. doi: 10.1186/s13046-023-02883-y (PMC10683265; doi:10.1186/s13046-023-02883-y)
Supplement: Supplementary file 1 — Additional file 1: Supplementary Table 1. cDNA primer sequences. Supplementary Table 2. Genomic DNA primer sequences. Supplementary Table 3. Genetic targeting of NR5A2. [file 13046_2023_2883_MOESM1_ESM.docx]

***Supplementary Table 1 – cDNA primer sequences***

| **Target gene** | **Fw sequence** | **Rv Sequence** |
| --- | --- | --- |
| *POU5F1* | AGAACATGTGTAAGCTGCGG | GGTTCGCTTTCTCTTTCGGG |
| *NANOG* | CCTGTGATTTGTGGGCCTGA | TGCGACACTCTTCTCTGCAG |
| *KLF4* | ACCCACACAGGTGAGAAACC | ATGTGTAAGGCGAGGTGGTC |
| *NODAL* | AGCATGGTTTTGGAGGTGAC | CCTGCGAGAGGTTGGAGTAG |
| *NR5A2* primer #1 | CCTTCCCAAGGCCACGAAAT | TTGAGACACAATAGGTGTAAGTCCG |
| *NR5A2* primer #2 | GGGTACCATTATGGGCTCCT | TGTCAATTTGGCAGTTCTGG |
| *TUBB* | ATGTTCCTCGTGCCATCCTG | CTGCCCCAGACTGACCAAAT |
| *SOX2* | AGAACCCCAAGATGCACAAC | CGGGGCCGGTATTTATAATC |
| *HPRT* | CCTGGCGTCGTGATTAGTGAT | AGACGTTCAGTCCTGTCCATAA |
| *MYC* | CCCGCTTCTCTGAAAGGCTCTC | CTCTGCTGCTGCTGCTGGTAG |
| *PPARGC1A (*PGC1a*)* | TGACTGGCGTCATTCAGGAG | CCAGAGCAGCACACTCGA |
| *bACTIN* | GCGAGCACACGAGCCTCGCC | CATCATCCATGGTGAGCTGGCGG |

***Supplementary Table 2 – Genomic DNA primer sequences***

| **Target gene** | **Fw sequence** | **Rv sequence** |
| --- | --- | --- |
| *SOX2* | GGCTTTGTTTGACTCCGTGT | TCCCATTGTCCCGACGTAAA |
| *NR5A2* | AGGGGTATGTCACAAGCCAA | CTCAGAAGTGGAGAGGTGCT |
| *CDKN1A* (p21) | GCTGGGATCTGATGCATGTG | ACTTCTAGCTCACCACCACC |
| *MYC* | AGAGTTCTTTCACGGGCAGA | AAGTCCCAGAATCCACAGCA |
| Intergenic  (negative control) | CCTGCTGCCATTCACGTTAG | GACCTACTTGAGACTGGGCA |

***Supplementary Table 3 – Genetic targeting of NR5A2***

| **Target gene** | **Sequence** |
| --- | --- |
| *NR5A2* sh#1 | GCTGGACTACACAATGTGTAA |
| *NR5A2* sh#2 | CGAACTCTCGTTGATCAAA |
| *NR5A2* si#1 | ACGCATGTTAATCTATGCAA |
| *NR5A2* si#2 | ACGACAGGAGCTGATAAGCAA |
