# Supplementary material for: Inhibiting NR5A2 targets stemness in pancreatic cancer by disrupting SOX2/MYC signaling and restoring chemosensitivity
Source: J Exp Clin Cancer Res. 2023 Nov 28;42:323. doi: 10.1186/s13046-023-02883-y (PMC10683265; doi:10.1186/s13046-023-02883-y)
Supplement: Supplementary file 2 — Additional file 2: Figure S1. NR5A2 is overexpressed in pancreatic cancer stem cells. Figure S2. NR5A2 regulates proliferation of differentiated PDAC cells. Figure S3. NR5A2 controls stemness in PDAC. Figure S4. Inhibition of NR5A2 specifically eliminates tumor-initiating CSCs. Figure S5. NR5A2 promotes stemness by diminishing MYC expression. Figure S6. NR5A2 inhibition targets CSCs in vivo and extends survival in preclinical PDAC models. [file 13046_2023_2883_MOESM2_ESM.pdf]

Figure S1 – *NR5A2* is overexpressed in pancreatic cancer stem cells

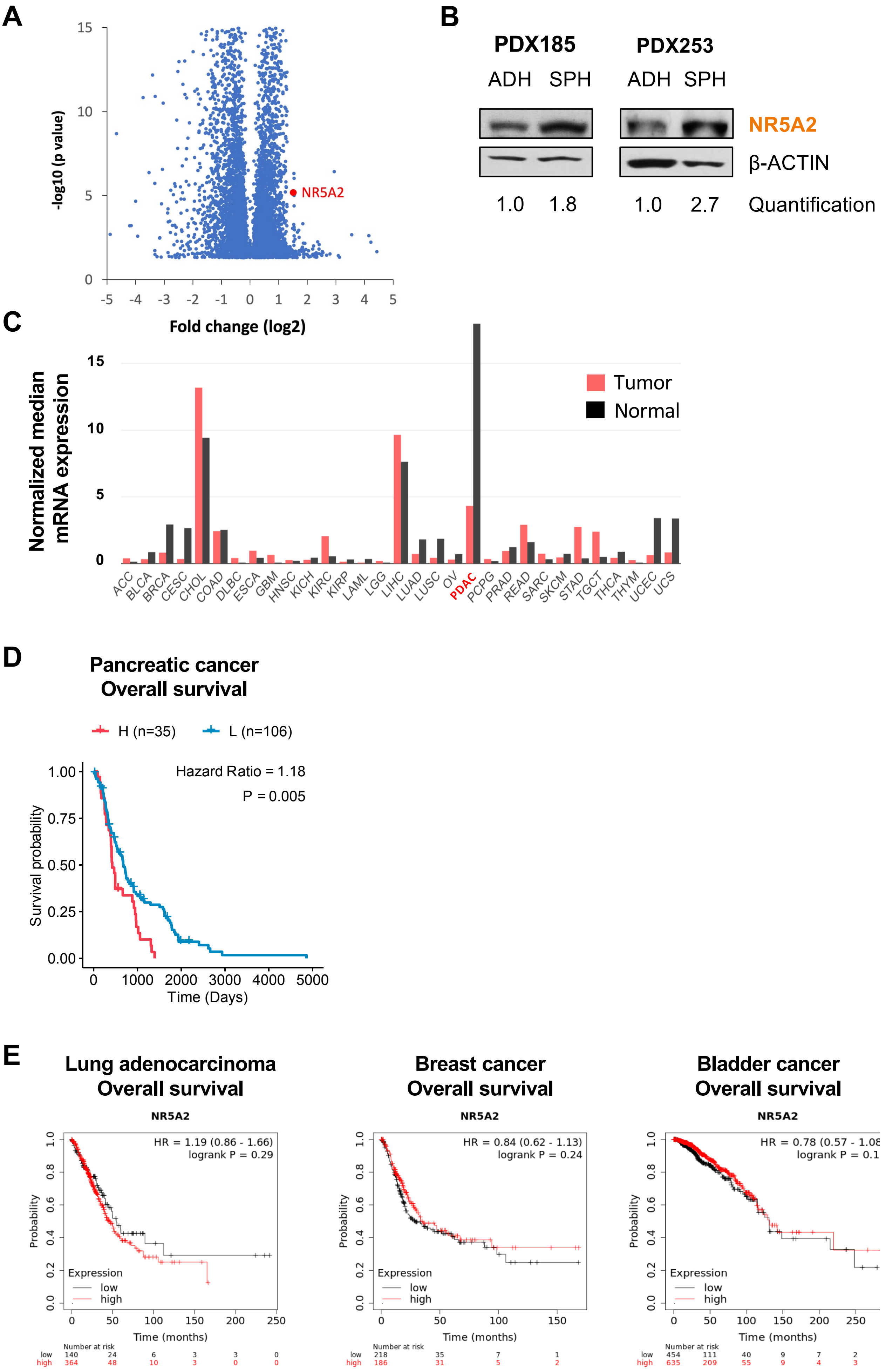

Figure S1 – *NR5A2* is overexpressed in pancreatic cancer stem cells

F

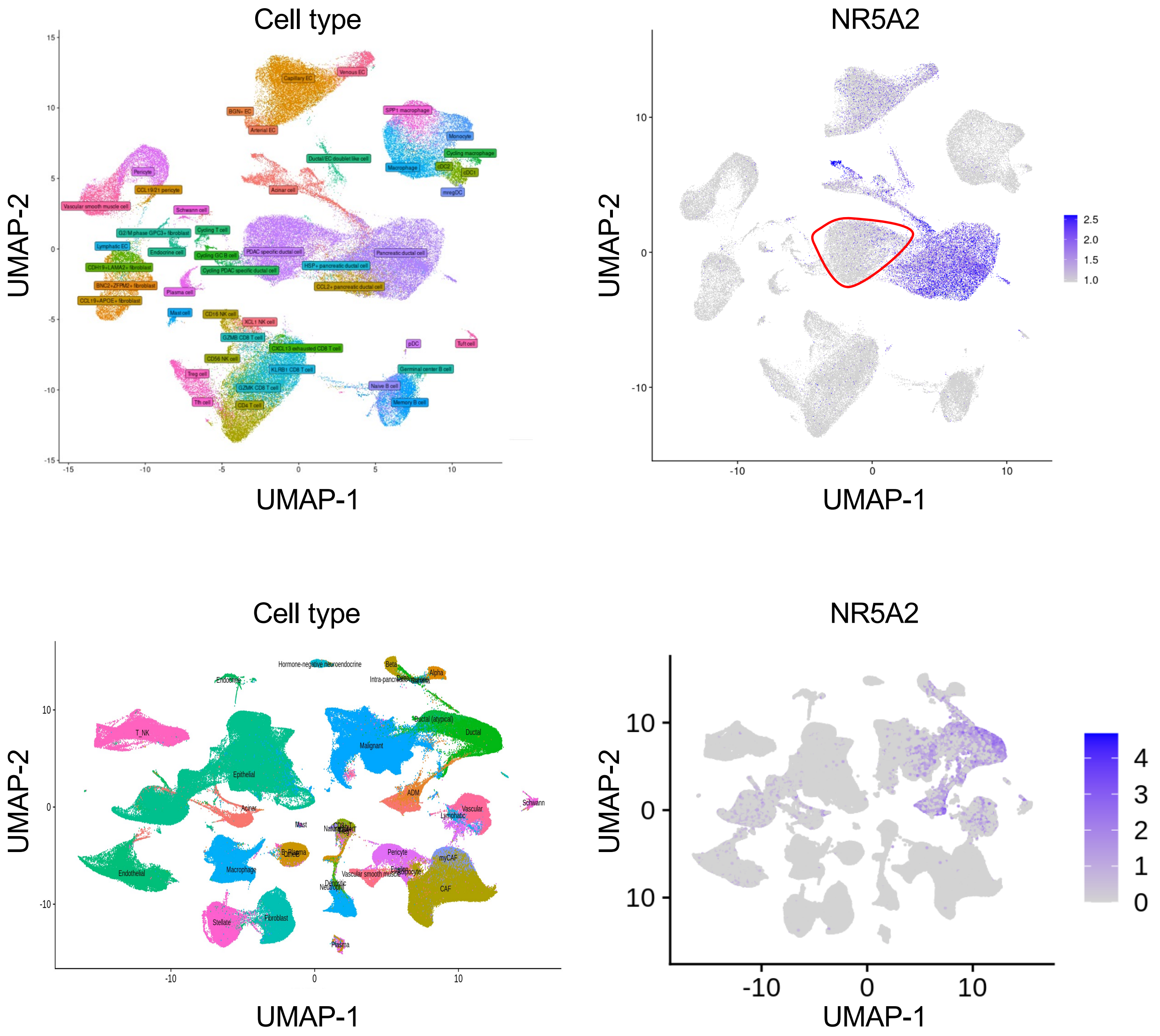

Figure S1 – *NR5A2* is overexpressed in pancreatic cancer stem cells

(**A**) Volcano plot of the gene expression changes between adherent and sphere cultures. (**B**) Western blot analysis comparing NR5A2 protein levels in adherent (ADH) versus sphere (SPH) cultures in PDX185 and PDX253 PDAC cultures. β-Actin was used as the loading control. (**C)** *NR5A2* mRNA expression across a large panel of human cancer and normal tissues using the GEPIA 2 database (http://gepia.cancer-pku.cn/index.html). ACC, Adrenocortical carcinoma; BLCA, Bladder Urothelial Carcinoma; BRCA, Breast invasive carcinoma; CESC, Cervical squamous cell carcinoma and endocervical adenocarcinoma; CHOL, Cholangiocarcinoma; COAD, Colon adenocarcinoma; DLBC, Lymphoid Neoplasm Diffuse Large B-cell Lymphoma; ESCA, Esophageal carcinoma; GBM, Glioblastoma multiforme; HNSC, Head and Neck squamous cell carcinoma; KICH, Kidney Chromophobe; KIRC, Kidney renal clear cell carcinoma; KIRP, Kidney renal papillary cell carcinoma; LAML, Acute Myeloid Leukemia; LGG, Brain Lower Grade Glioma; LIHC, Liver hepatocellular carcinoma; LUAD, Lung adenocarcinoma; LUSC, Lung squamous cell carcinoma; OV, Ovarian serous cystadenocarcinoma; PAAD, Pancreatic adenocarcinoma; PCPG, Pheochromocytoma and Paraganglioma; PRAD, Prostate adenocarcinoma; READ, Rectum adenocarcinoma; SARC, Sarcoma; SKCM, Skin Cutaneous Melanoma; STAD, Stomach adenocarcinoma; TGCT, Testicular Germ Cell Tumors; THCA, Thyroid carcinoma; THYM, Thymoma; UCEC, Uterine Corpus Endometrial Carcinoma; UCS, Uterine Carcinosarcoma. (**D**) Prognostic significance of NR5A2 mRNA expression levels for overall survival of pancreatic cancer patients as analyzed by R programming language using the ArrayExpress database E-MTAB-1791 (Jandaghi P et al. Gastroenterology. 2016 Dec;151(6):1218-1231). **(E**) Prognostic significance of *NR5A2* mRNA expression levels for overall survival for patients with different cancer types as analyzed by Kaplan-Meier Plotter (https://kmplot.com/analysis/). (**F**) Deeply Integrated human Single-Cell Omics data (Li, Mengwei, et al. "DISCO: a database of Deeply Integrated human Single-Cell Omics data." Nucleic acids research 50.D1 (2022): D596-D602; https://www.immunesinglecell.org) were used to analyze RNA-seq data from the PDAC v1.0 disease atlas, which included 48 different cell types and 168K cells (**left panel**). UMAP plots were generated to visualize the expression of NR5A2 in specific cell types, including PDAC-specific ductal cells (red circle: **right panel**).

Figure S2 – *NR5A2* regulates proliferation of differentiated PDAC cells

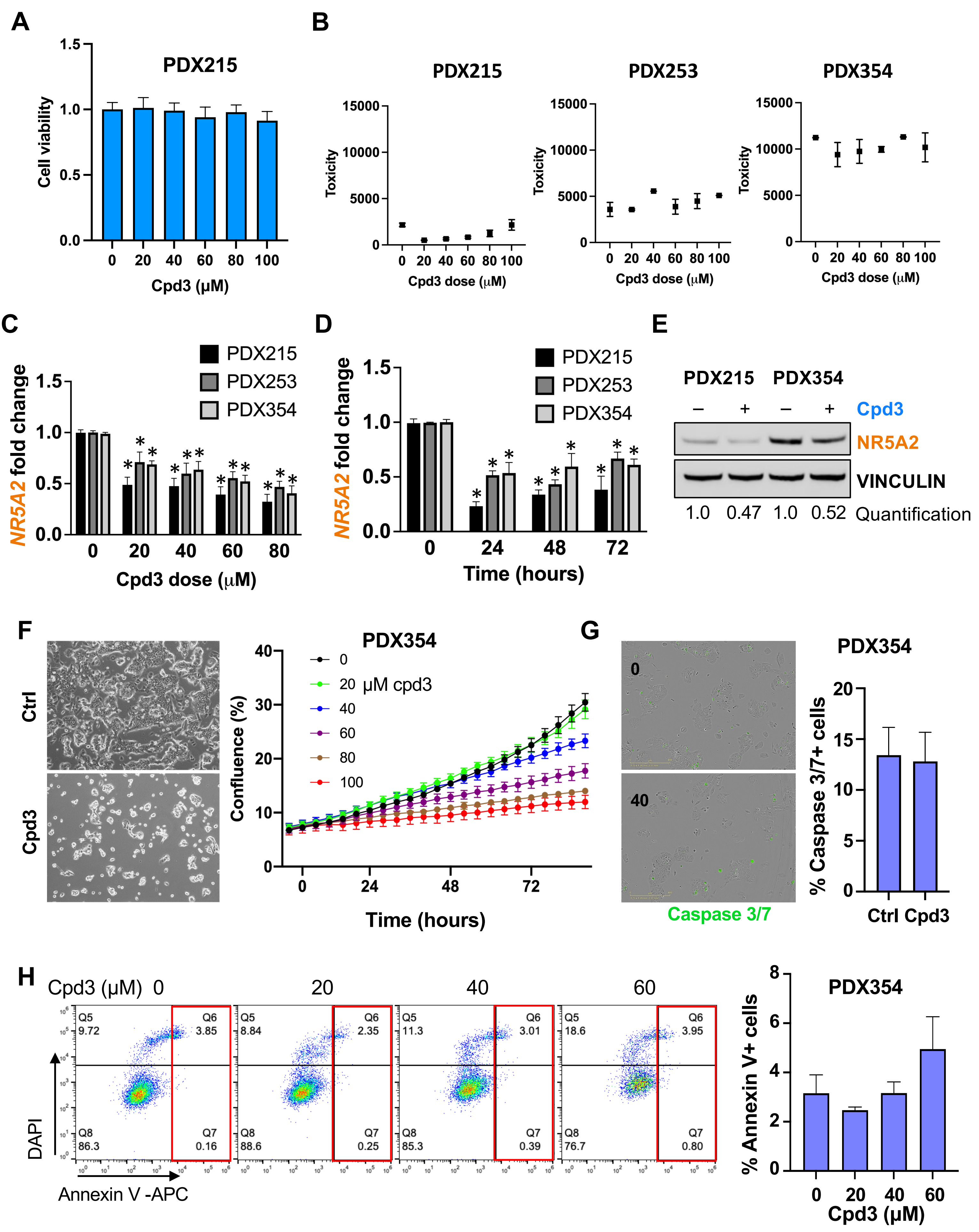

Figure S2 – *NR5A2* regulates proliferation of differentiated PDAC cells

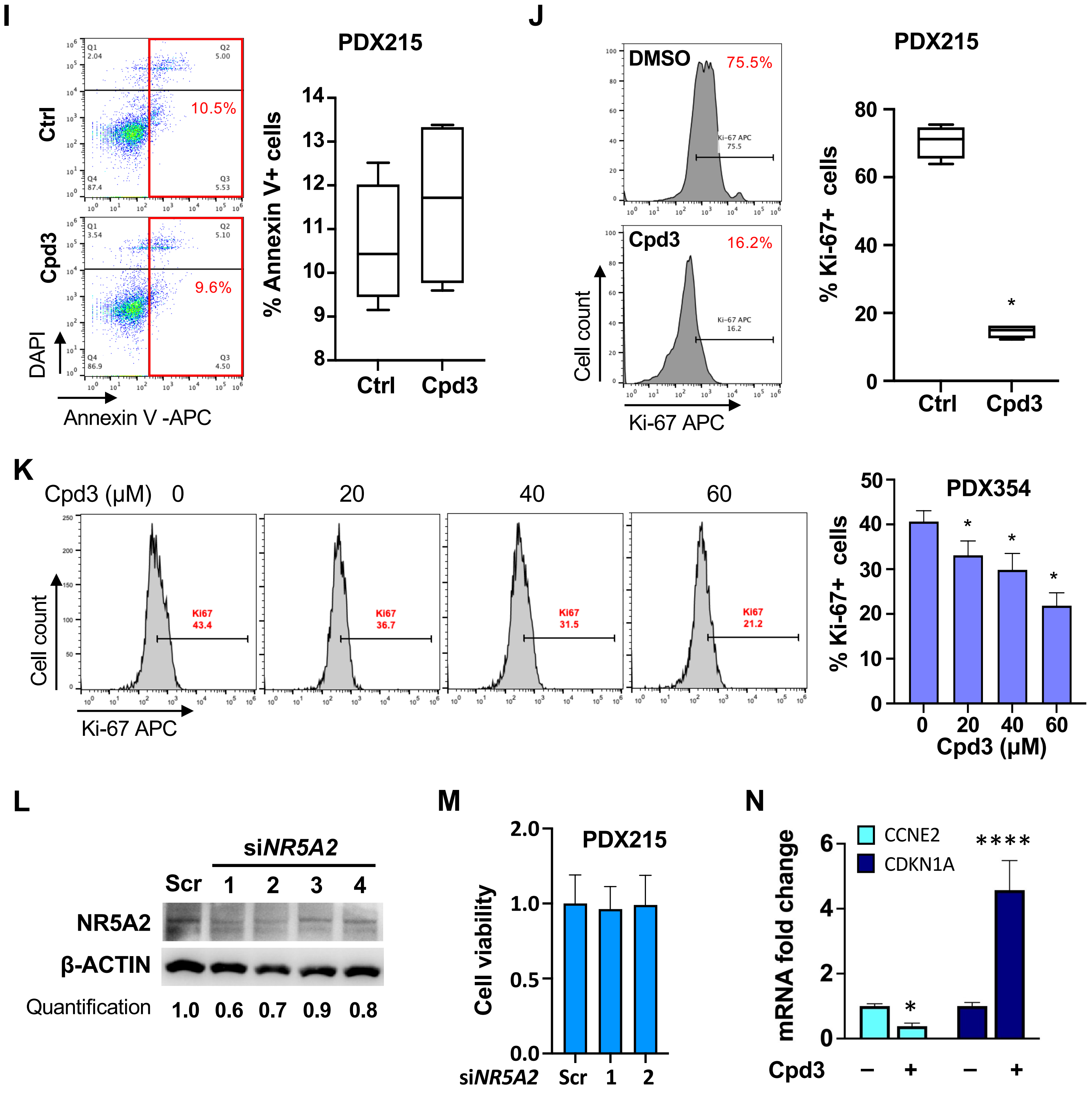

Figure S2 – *NR5A2* regulates proliferation of differentiated PDAC cells

(**A**) Cell viability was assessed by quantifying the number of viable cells in culture using a luminescent measurement of ATP levels. Graded doses of Cpd3 were tested for 24 hours to evaluate their effects on cell viability. (**B**) Adenylate kinase (AK) release-based toxicity in media following Cpd3 treatment at increasing concentrations at 24 hours. (**C**) The qPCR fold change for *NR5A2* mRNA using three different PDAC cultures exposed to increasing concentrations of the NR5A2 inhibitor Cpd3 for 72 hours (n=4 biological replicates). (**D**) The fold change in *NR5A2* mRNA levels was determined by qPCR at 24 hours, 48 hours, and 72 hours after a single-shot treatment with 80 µM Cpd3. (**E**) Western blot analysis of NR5A2 protein levels at 72 hours following treatment with Cpd3 in PDX215 and PDX354 PDAC cultures. Vinculin was used as a loading control. (**F**) Cell density and morphology after 72 hours of treatment with 40 µM Cpd3 (**left panel**). Overall confluency was followed over a period of 80 hours using the Incucyte platform (**right panel**). Representative results for PDX354 from n=5 experiments. (**G**) Caspase 3/7 staining was performed on cells treated with control or 40 µM Cpd3 for 72 hours. Representative images depicting the staining in PDX354 cells, along with corresponding quantification, are shown. (**H**) Apoptosis analysis was conducted using DAPI/Annexin V flow cytometry in PDX354 cells treated with graded doses of Cpd3. The lower right quadrant indicates early apoptosis, while the upper right quadrant represents late apoptosis (marked by the red rectangle). Quantification reveals the percentage of combined early and late apoptotic cells (n=3). (**I**) Similarly, apoptosis analysis was performed in PDX215 cells treated with 80 µM Cpd3 for 72 hours (n=4). (**J**) Flow cytometric analyses were carried out to examine Ki-67 expression after treatment with 80 µM Cpd3 for 72 hours in PDX354 cells. The quantification depicts the percentage of Ki-67+ cells (n=4). (**K**) Correspondingly, proliferation analysis assessing the number of Ki-67+ cells was conducted in PDX354 cells treated with graded doses of Cpd3. Representative flow cytometry histograms (**left**), quantification of n=3 biological replicates is shown (**right**). (**L**) Efficacy of si*NR5A2* in suppressing NR5A2 protein levels was assessed by Western blot. Control (scr: scramble siRNA) and four different siNR5A2 (#1 to #4) were tested for 72 hours. β-Actin was used as the loading control. (**M**) Cell viability was assessed by quantifying the number of viable cells in culture using a luminescent measurement of ATP levels. siRNA were tested for 24 hours to evaluate their effects on cell viability. The panel displays the effects of scramble siRNA and the two most effective siNR5A2 variants (#1 and #2) on cell viability. (**N**) qPCR fold change of *CCNE2* and *CKDN1A* (p21) mRNA levels following 72-hour treatment with 80 µM Cpd3. * $p<0.05$, **** $p<0.0001$; Statistical analysis was performed using the Mann-Whitney test.

Figure S3 – *NR5A2* controls stemness in PDAC

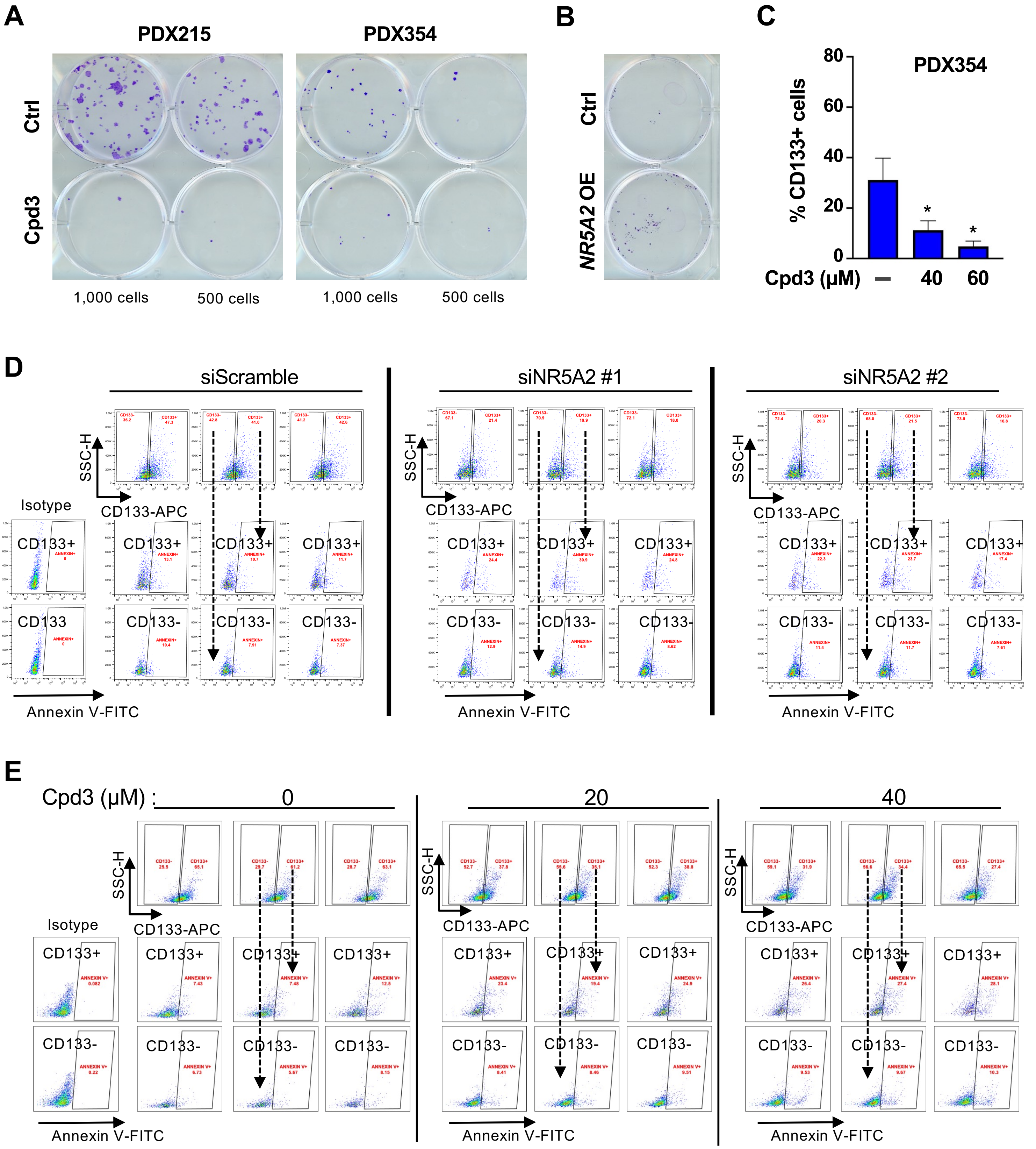

Figure S3 – *NR5A2* controls stemness in PDAC

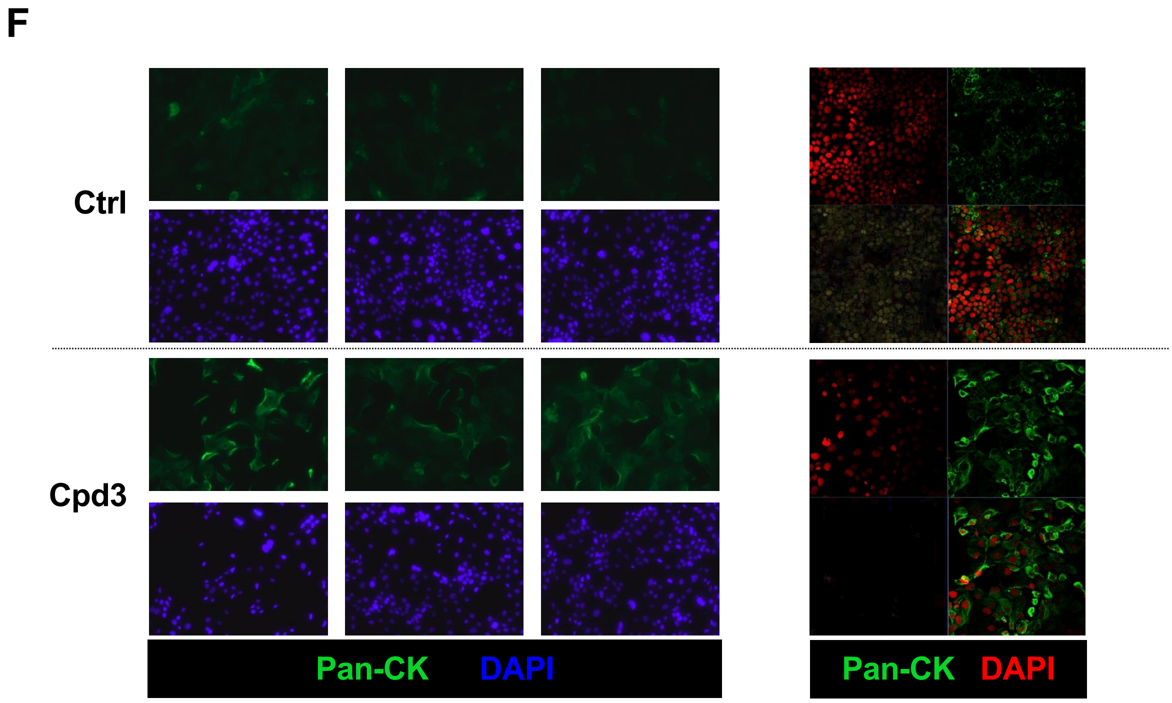

(**A**) Colony formation capacity of PDAC cells treated with Cpd3 for 72 hours. (**B**) Colony formation capacity of PDAC cells overexpressing *NR5A2*. **(C**) Flow cytometry analysis of the CSC marker CD133 in second-generation spheres in the presence of Cpd3 in PDX354 cells. (**D**) Flow cytometry analysis of Annexin V in second-generation spheres sorted into CD133+ and CD133- cells, treated with two different siNR5A2 variants, or 20 and 40 µM Cpd3 in PDX215 cells (**E**). (**F**) Immunofluorescence for Pan-cytokeratin (green) following 48 hours of treatment with 80 µM Cpd3. Nuclei were stained with DAPI (blue and red, respectively). * p<0.05; Statistical analysis was performed using the Mann-Whitney test.

Figure S4 – Inhibition of *NR5A2* specifically eliminates tumor-initiating CSCs

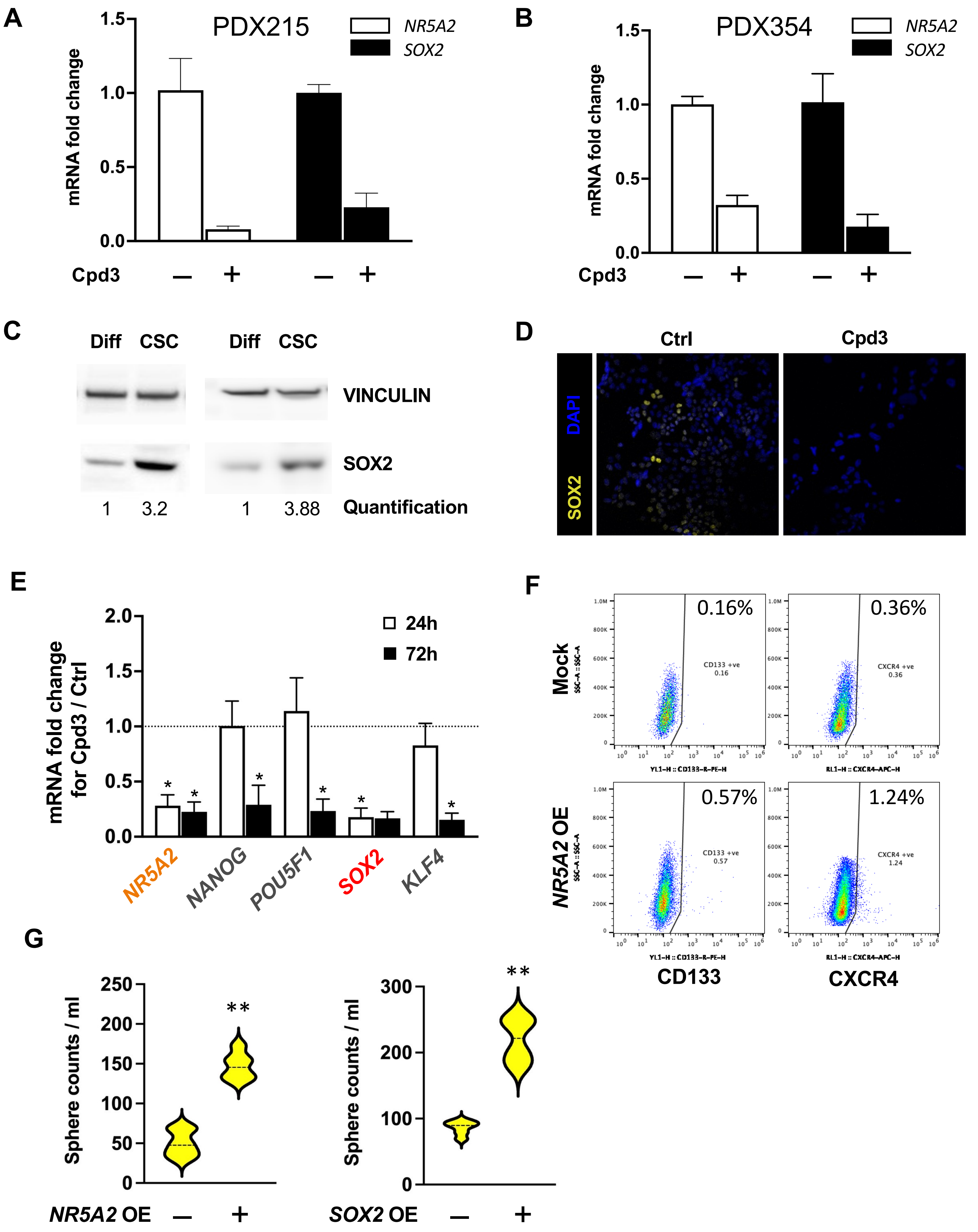

Figure S4 – Inhibition of *NR5A2* specifically eliminates tumor-initiating CSCs

H

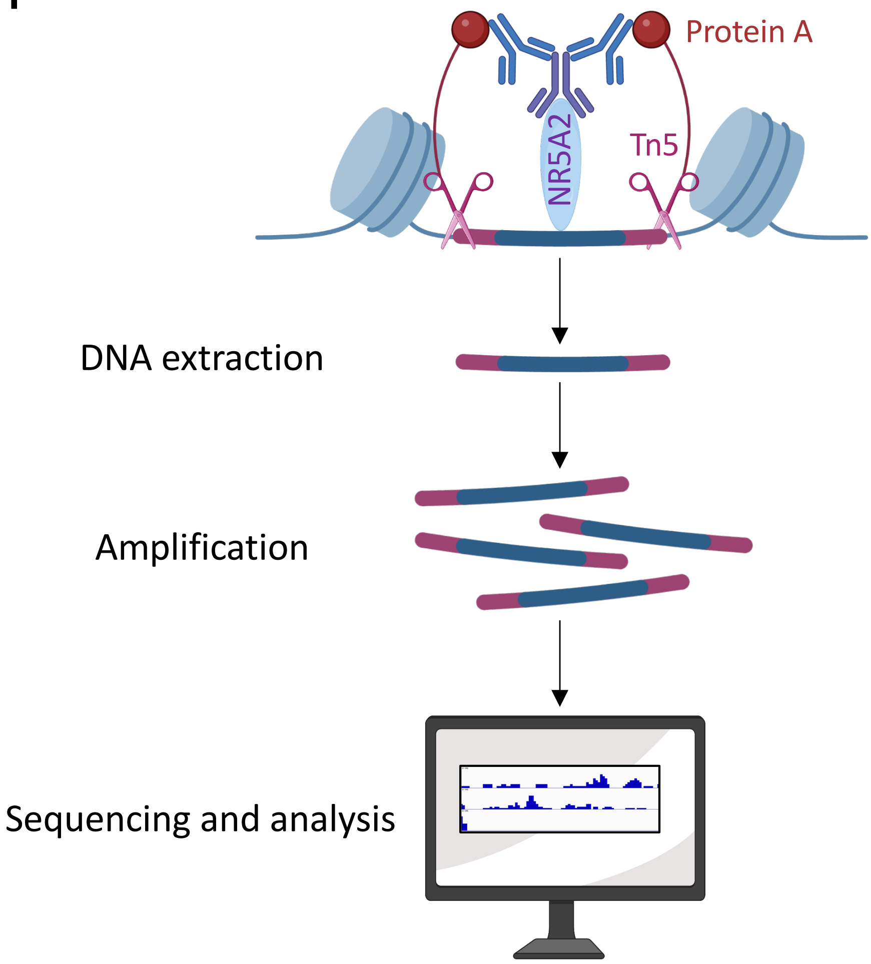

Quantitative PCR fold change of *NR5A2* and *SOX2* mRNA levels following 72-hour treatment with Cpd3 in PDX215 (**A**), and in PDX354 cells (**B**). (**C**) Western blot analysis of SOX2 protein levels in adherent (differentiated, Diff) versus sphere (CSC) cultures in two different PDAC models. Quantification by Image J analysis. (**D**) Immunofluorescence for SOX2 (yellow) following control (Ctrl) DMSO (**left**) or Cpd3 (**right**) treatment (40 µM) for 72 hours. Nuclei are stained with DAPI (blue). (**E**) RNA levels for stemness-associated genes following Cpd3 treatment (40 µM) at 24 hours and 72 hours. The dotted line indicates baseline expression levels, set as 1.0. **(F)** Flow cytometry for CD133 and CXCR4 expression in sorted $CD133^{-}FLUO^{-}$ differentiated cancer cells following overexpression of *NR5A2*. (**G**) Sphere formation capacity using sorted $CD133^{-}FLUO^{-}$ differentiated cancer cells following overexpression of *NR5A2* or *SOX2.* The dashed lines in the violin plots indicate the median. (**H**) Simplified schematic of the CUT&TAG procedure for investigating protein-DNA interactions. Native unfixed cells are incubated with a primary antibody targeting the NR5A2 protein (purple) located between nucleosomes in the genome, followed by the addition of a secondary antibody (blue). Protein A fused to the Tn5 transposase enzyme is then introduced. Upon activation of protein A-Tn5, cleavage of intact DNA and insertion of adapters (tagmentation, shown in red) occurs for paired-end DNA sequencing. After DNA purification, genomic fragments with adapters at both ends are enriched by PCR. Subsequently, sequencing and analysis are performed to identify NR5A2-DNA interaction sites. * $p<0.05$ and ** $p<0.01$; Statistical analysis was performed using the Mann-Whitney test [created with Biorender.com].

Figure S5 – *NR5A2* promotes stemness by diminishing *MYC* expression

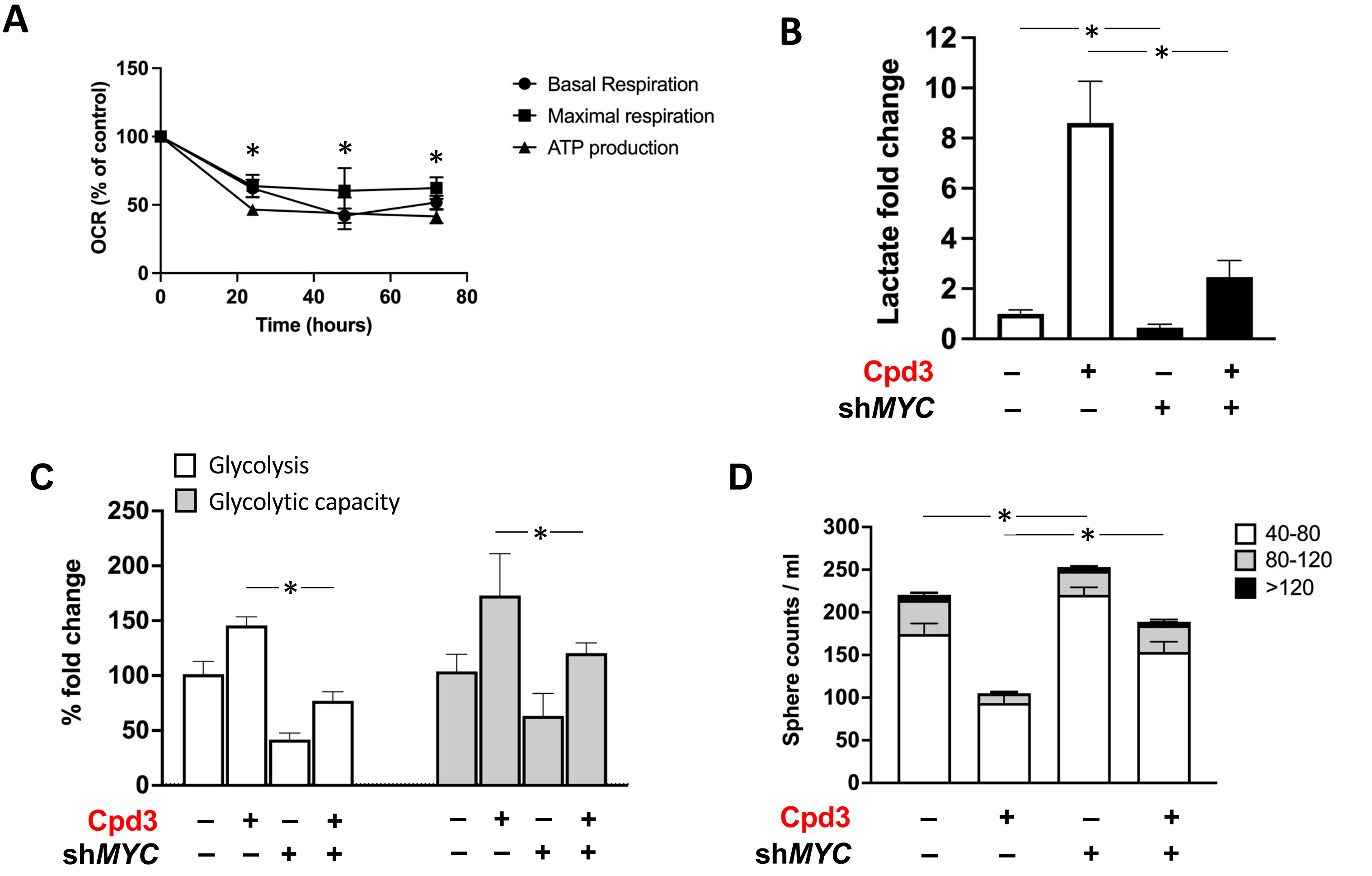

(**A**) Oxygen consumption rate (OCR) changes for basal respiration, maximal respiration, and ATP production following treatment with Cpd3 for 24, 48, and 72 hours in spheres. (**B**) Lactate production in shNT and shMYC cells following 72-hour treatment with DMSO (Ctrl) or Cpd3. (**C**) Measurement of glycolysis (white) and glycolytic capacity (grey) in shNT and shMYC cells following 72-hour treatment with DMSO (Ctrl) or Cpd3. (**D**) Assessment of sphere formation capacity in cells with shNT or shMYC following 72-hour treatment with DMSO (Ctrl) or Cpd3. * $p<0.05$; Statistical analysis was performed using the Mann-Whitney test.

Figure S6 – NR5A2 inhibition targets CSCs in vivo and extends survival in preclinical PDAC models.

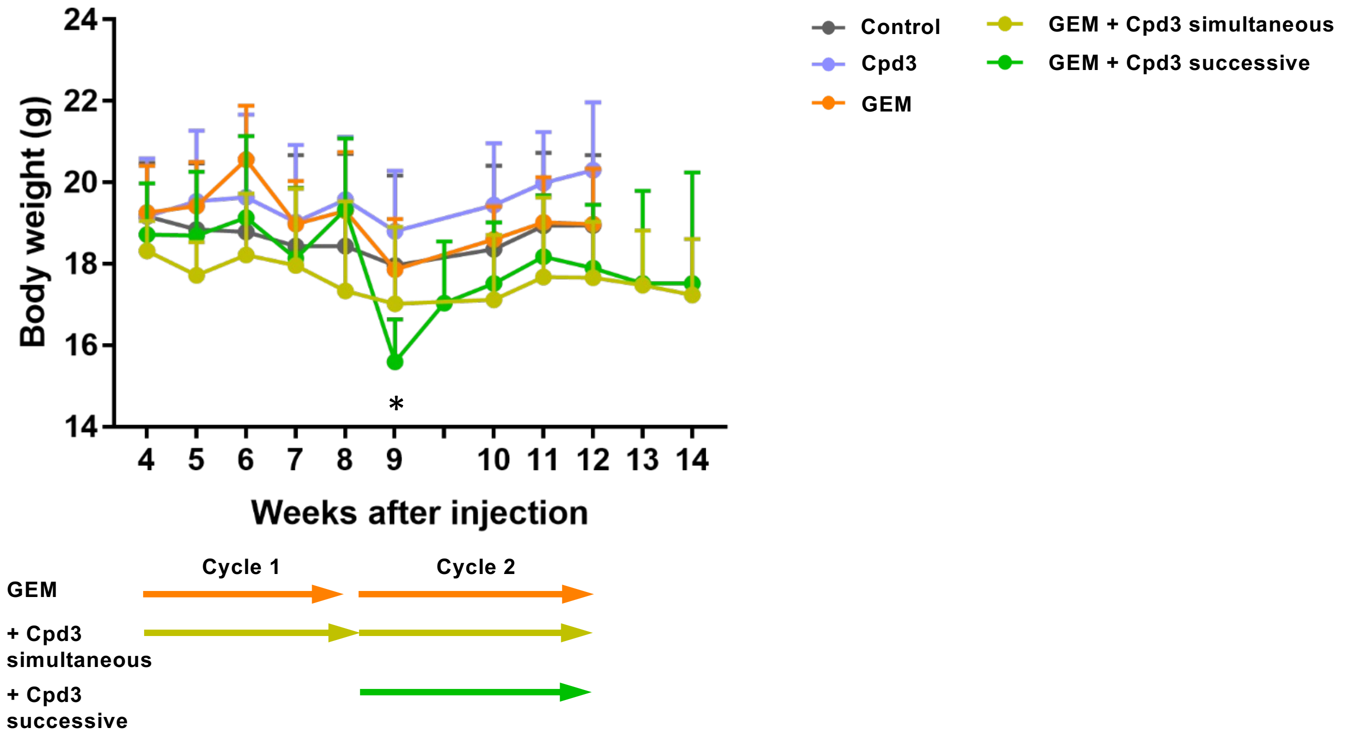

Body weight according to allocated treatments with two treatment cycles of 28 days each.
* $p<0.05$; Statistical analysis was performed using the Mann-Whitney test.
